# Did the UK policy response to Covid-19 protect household incomes?

**DOI:** 10.1007/s10888-021-09491-w

**Published:** 2021-08-06

**Authors:** Mike Brewer, Iva Valentinova Tasseva

**Affiliations:** 1 Resolution Foundation, 2 Queen Anne’s Gate, London, SW1H 9AA UK; 2 London School of Economics and Political Science, Houghton Street, London, WC2A 2AE UK

**Keywords:** Covid-19, Earnings subsidies and tax-benefit policies, Income distribution, D31, E24, H24

## Abstract

We analyse the UK policy response to Covid-19 and its impact on household incomes in the UK in April and May 2020, using microsimulation methods. We estimate that households lost a substantial share of their net income of 6.9% on average. But policies protected household incomes to a substantial degree: compared to the drop in net income, GDP per capita fell by 18.9% between the first and second quarter of 2020. Earnings subsidies (the Coronavirus Job Retention Scheme) protected household finances and provided the main insurance mechanism during the crisis. Besides subsidies, Covid-related increases to state benefits, as well as the automatic stabilisers in the tax and benefit system, played an important role in mitigating the income losses. However, analysing the impact of a near-decade of austerity on the UK safety net, we find that, compared to 2011 policies, the 2020 *pre-Covid* tax-benefit policies would have been less effective in insuring incomes against the shocks. We also assess the potential distributional impact of introducing a Universal Basic Income (UBI) instead of the Covid emergency measures and find that a UBI would have supported the incomes of different vulnerable groups but would have provided less protection to those hit hardest by the labour market shocks.

**Supplementary Information:**

The online version contains supplementary material available at 10.1007/s10888-021-09491-w.

**JEL codes** D31 · E24 · H24

✉ Iva Valentinova Tasseva
i.tasseva@lse.ac.uk

Mike Brewer
mike.brewer@resolutionfoundation.org

## 1 Introduction

The UK has been one of the hardest hit countries in the world by the Covid-19 pandemic. At the time of writing, the UK is in top 20 and top 5 globally for the highest total number of infected cases and deaths per million people, respectively.[1] To slow down the spread of the disease, the UK government introduced strict lockdown measures from 23 March 2020 until the end of May 2020 closing down economic sectors such as hospitality and non-essential retail and restricting severely people's movements. This sharp drop in activity meant that the UK has been among the most adversely-affected economically by the pandemic among OECD countries. For example, UK GDP per capita fell by 18.9% and household consumption expenditure fell by 22.4% in the second quarter of 2020, compared to the first – the highest recorded drops in the OECD.[2]

To minimise the damage to families staying at home and the economy, the UK government designed at speed a large emergency package consisting of two brand-new programmes for employees, the *Coronavirus Job Retention Scheme (CJRS)*, and for the self-employed, the *Self-Employment Income Support Scheme (SEISS)*, as well as increasing the generosity of state benefits for low-income families (*Universal Credit* and other benefits). This paper assesses the impact of the Covid-19 crisis and these emergency reforms on household incomes during the months of the strictest lockdown measures and the highest recorded drop in GDP since the start of the pandemic – April and May 2020. It aims to provide a better understanding of the distributional impact of the existing and new fiscal policies introduced by the government. Furthermore, we analyse how austerity measures over the last decade, in terms of cuts to state benefits for low-income families, have affected the UK safety net. We also assess what would have been the distributional impact of the pandemic if a Universal Basic Income (UBI) was introduced in place of the Covid emergency measures.

As survey micro-data on household incomes during the pandemic will only become available with a few years' lag, the present paper combines different data sources and a tax-benefit model to predict household circumstances in April and May 2020. In more detail, we construct a baseline income distribution using data from the Family Resources Survey (FRS) of 2018/19. Then, using individual-level data from the Understanding Society COVID-19 Study on employment and earnings changes between February and April/May 2020, we simulate similarly-sized employment and earnings shocks on the sample of workers from the FRS. Workers affected by the shocks become out of work or furloughed, or stay employed (not furloughed) but their hours and earnings fall. Furloughed workers receive earnings subsidies from the CJRS while self-employed can receive support from the SEISS. Using the tax-benefit model UKMOD, we then calculate household income tax liabilities and benefit entitlements before and after the employment and earnings shocks. The changes in income taxes and state benefits due to the shocks capture the automatic stabilisation response of the tax-benefit system before the Covid-related benefit increases (Auerbach and Feenberg 2000). Finally, we estimate the effect of Covid-related increases to state benefits on household incomes, holding constant the earnings distribution after the shocks.

The Covid-19 pandemic hit hard household incomes. We simulate that the loss in household net income was substantial of 6.9% on average. But policies protected household incomes

[1] Our World in Data accessed on 7 February 2021, https://ourworldindata.org/coronavirus.

[2] OECD, Household Dashboard accessed on 21 April 2021, https://www.oecd.org/sdd/na/household-dashboard.htm.

to a substantial degree. Compared to the drop in net income, GDP per capita fell by 18.9% between the first and second quarter of 2020. Moreover, we estimate that the emergency policies – CJRS, SEISS and increases to means-tested benefits – lowered income losses by substantial 9.3 percentage points (pp) on average, assuming that all furloughed workers would have been made unemployed in the absence of the emergency measures.

In more detail, the CJRS for furloughed workers in particular protected household finances from the shocks, accounting for 13.2% of baseline net income. The CJRS supported households across the entire income distribution and provided the main insurance mechanism against the negative income shocks. The SEISS also contributed to income gains at all parts of the distribution, of 1.3% on average. Net of the subsidies, the loss in earnings from both employment and self-employment amounted to a substantial 12.6% of net income.

Besides the earnings subsidies, the Covid-related increases to state benefits and tax-benefit automatic stabilisers also had an important role in mitigating income losses. Comparing the impact of the benefit increases and automatic stabilisers on net incomes, it is the latter that had the bigger effect on net incomes, underlining the importance of tax-benefit designs in protecting household incomes during economic downturn. On the other hand, different parts of the distribution rely on different types of policy for mitigating income shocks, stressing the importance of both automatic stabilisers and governments' responses to crises in determining the amount of income protection.

Assessing the impact of a near-decade of austerity on the UK safety net, we show that, compared to the 2011 policies, the 2020 *pre-Covid* tax-benefit policies would have been less effective in providing insurance against the shocks for all parts of the distribution. The extra Covid benefit spending strengthened the UK safety net, so that compared to the 2011 system the 2020 *post-Covid* system provided higher levels of income protection in the bottom half of the distribution. But although the Covid-related benefit increases strengthened the safety net, at the time of writing these emergency measures are due to expire in September 2021.

Finally, we find that there would have been different gainers and losers from the introduction of a UBI in place of the Covid emergency measures. A UBI would have provided less protection, compared to the emergency measures, to those hit hardest by the labour market shocks. On the other hand, different vulnerable groups such as households with no earners, elderly or disabled individuals would have gained from a UBI.

This paper relates to several strands of literature. It relates to the now fast-growing literature on the economic and distributional impact of Covid-19. Using the Understanding Society COVID-19 data, Crossley et al. (2021) analyse the economic impact of the pandemic in the UK on employment and earnings, and the ways different household types mitigate the negative shocks, highlighting the importance of transfers from family and friends. Using timely data on the US, Han et al. (2020) assess the impact of the coronavirus crisis and increases to government assistance on US poverty. Adams-Prassl et al. (2020) assess differences in the labour market impact of Covid-19 in the UK, Germany and US. Brewer and Gardiner (2020) summarise what was known about the distributional impact of the crisis in the UK as of September 2020, focusing in particular on low-income households. By simulating employment shocks and using a tax-benefit model, Bruckmeier et al. (2020), Figari and Fiorio (2020), Beirne et al. (2020) and Bronka et al. (2020) assess the distributional impact of Covid-19 in Germany, Italy, Ireland and the UK, respectively. In particular, the paper by Bronka et al. (2020) uses a consensus analysis based on economists' opinions about how different parts of the economy would be affected by the pandemic, rather than actual data on how the crisis had affected employment patterns.

The current paper builds on and contributes to the existing literature in several ways. The paper brings new insights into the importance of different types of policy in mitigating income losses along the income distribution and quantifies the income compensation provided by the policy response. It also examines empirically how policy reforms over the last decade have reduced the ability of the UK safety net to cushion negative economic shocks and how effective a UBI would have been in protecting household incomes instead of the emergency measures. As such, it also contributes to the evidence on the mitigating role of tax-benefit policies during recessions. Income taxes and unemployment insurance benefits have been found to be particularly important in providing income protection and income and consumption smoothing during recessions (Larrimore et al. 2015; Fernández Salgado et al. 2014; Dolls et al. 2012; Kniesner and Ziliak 2002a, 2002b; Auerbach and Feenberg 2000). Furthermore, means-tested benefits have been shown to be effective in providing a much-needed safety net for households during economic downturns (Bitler et al. 2017; Bitler and Hoynes 2016). Recent analysis has also highlighted the importance of tax and benefit automatic stabilisers for income redistribution (Paulus and Tasseva 2020).

The rest of the paper is structured as follows: Section 2 describes the UK policy response to Covid-19 and the tax-benefit policies that played a role in protecting household incomes from the negative income shocks. Section 3 discusses the decomposition approach to identify the effect of earnings subsidies, tax-benefit automatic stabilisers and Covid-related benefit increases; the data and the simulation of employment and earnings shocks; and the tax-benefit model UKMOD. Section 4 discusses the results and section 5 concludes.

## 2 The UK policy response to Covid-19 and tax-benefit policies

During the Covid-crisis, access to unemployment and income-related benefits as well as income taxes and national insurance contributions provide insurance against the economic shocks. In addition, the UK government introduced a package of policy measures in response to Covid-19. These include income protection schemes for workers and increases to state benefits. This section describes the Covid-related policy measures, as well as discussing which tax-benefit policies play a role in stabilising household incomes during crisis.

To support businesses and workers, the Coronavirus Job Retention Scheme (CRJS) was introduced to subsidise the earnings of furloughed employees. This allows companies to reduce the hours of workers to zero without laying them off, removing the costs of searching and re-hiring workers later on. In April and May, the CJRS paid 80% of gross earnings up to a maximum of £2500 per month. Similarly, the Self-Employment Income Support Scheme (SEISS) was introduced to provide support for self-employed affected by the pandemic. The scheme was opened for claims on May 12. It is calculated based on the person's average trading profit over the 3 previous tax years and equals 80% of the average profit, paid in a single instalment covering three-months worth of profit, up to £7500 in total.

In addition to the earnings subsidies, families affected by the economic shocks can access a range of state benefits to compensate for the losses in earned income. The main ones are the contribution-based unemployment benefit, Jobseeker's Allowance (JSA), and the means-tested benefit Universal Credit (UC). JSA is a flat-rate unemployment benefit paid up to 6 months while looking for a job. The benefit is available to those who have paid employees' national insurance contributions. It has two rates: for those aged under 25 (£58.9 per week) and a more generous rate for those aged 25+ (£74.35 per week). UC is an income-tested benefit for working-age people on low-incomes or who are unemployed. It was introduced in 2014 with the aim to gradually replace a range of income-related benefits and tax credits, by combining them into a single state transfer. The benefit consists of a basic 'standard allowance' and extra payments which depend on the person's and their family's circumstances. UC is withdrawn in line with the joint earnings of the family, and overall entitlement is subject to a maximum ceiling known as a benefit cap. Prior to Covid-19, the UC rate for singles aged 25+ was the same as JSA, £323 per month, while the rate for couples was about 1.6 times higher, £507 per month.

In response to the crisis, the UK government increased the level of UC for all family types by £20 a week. This is a substantial increase of 28% for singles aged 25+ and 17% for couples. Access to UC for the self-employed was also improved. Other Covid-related changes included increases to Housing Benefit (HB) and the UC component which support low-income families with paying their rent. The amount of earnings disregarded in calculating the entitlements to HB and Council Tax Reduction (a benefit that supports families with paying property tax) was also increased. As with UC, the standard allowance rates for Working Tax Credit – an in-work benefit and one of the benefits that UC is replacing – also went up by £20 a week. At the time of writing, these changes were due to expire in September 2021. For more details on the emergency measures and existing policies, see Reis and Tasseva (2020).

One contribution of this paper is to identify how the tax and benefit system automatically responds to cushion the impact of labour market shocks. As well as the benefit system, income tax and national insurance contributions (NIC) also have an important role to play in mitigating income losses. The income tax schedule in the UK is progressive, as are NIC for most parts of the distribution.[3] Thus, as earnings drop due to the economic shocks, income tax payments and NIC also decrease, so that after-tax income drops by less than pre-tax income.

## 3 Data and methodology

### 3.1 Estimating the distributional impact of the crisis

Our aim is to estimate the impact of the crisis on the income distribution using the methodology from Paulus and Tasseva (2020) and Bargain and Callan (2010) who combine household micro-data with tax-benefit microsimulation techniques. To provide a detailed picture of the effectiveness of different types of policy in protecting household incomes against the shocks, we decompose the changes in the income distribution to identify separately the impact of: earnings losses; earnings subsidies; tax-benefit policies before the Covid-related increases to state benefits (which we refer to as the automatic stabilisation response of policies); and the Covid-19 benefit increases.

[3] Employee and self-employed pay different rates of NIC but common rules apply to both types of worker. Broadly, workers on low incomes are exempt from NIC. Once they start earning above a certain minimum and up to an upper threshold, they pay a certain rate of NIC. Above the upper threshold, they pay a lower rate on the amount of earnings exceeding the threshold. Thus, for most parts of the distribution, the average rate of NIC increases with earnings, i.e. it is progressive; but once earnings exceed the upper threshold, NIC paid as a fraction of earnings start to fall.

### 3.1.1 Impact of the crisis

Let us denote with $y$ gross (pre-tax) market income; with $t$(y) income tax and NIC, which are a function of $y$; and with $b(t, y)$ state benefits which are a function of $t$ and $y$. Household net income B (for *baseline*) is then:

$$B = y - t(y) + b(t, y) \tag{1}$$

An economic shock occurs which raises unemployment or leads to some workers being furloughed, and lowers gross earnings. The UK government responded by introducing earnings subsidies for furloughed workers and increasing state benefits, so that gross market income after the shock ($y_k$) plus subsidies ($k$) is $y'$; income tax liabilities and NIC after earnings changes are $t(\mathrm{y}')$; and state benefits after earnings and benefit changes are $b''$ (t, y′). Household net income after the shock can be shown to be:

$$D = y' - t(\mathrm{y}') + b''(\mathrm{t}, \mathrm{y}') \tag{2}$$

and the difference between $D$ and $B$ gives the impact of the crisis on household incomes.

### 3.1.2 Decomposing changes in the income distribution

We can introduce an intermediate counterfactual scenario $C$ after the shock and earnings subsidies, but before the crisis-related benefit increases. This allows us to decompose the change in income Δ:

$$\Delta = D - B = \underbrace{D - C}_{\text{benefit policy changes}} + \underbrace{C - B}_{\text{earnings changes+automatic stabilisers}} = \underbrace{y' - t(\mathrm{y}') + b''(\mathrm{t}, \mathrm{y}') - (y' - t(\mathrm{y}') + b(\mathrm{t}, \mathrm{y}'))}_{\text{benefit policy changes}} + \underbrace{y' - t(\mathrm{y}') + b(\mathrm{t}, \mathrm{y}') - (y - t(\mathrm{y}) + b(\mathrm{t}, \mathrm{y}))}_{\text{earnings changes+automatic stabilisers}} \tag{3}$$

where the difference between $D$ and $C$ gives the effect of benefit increases ($P$), keeping fixed the distribution of gross market incomes, tax liabilities and NIC at their post-crisis level. The difference between $C$ and $B$ gives the total effect of earnings changes plus automatic changes to income tax, NIC and benefits, keeping fixed the tax-benefit rules at their pre-crisis levels (i.e. effect of automatic stabilisers).

Let us now denote as $I$ a function of income. If $I$ is additively decomposable by income source, e.g. mean net income, we can break down the difference between $C$ and $B$ further into earnings losses ($E$), earnings subsidies ($K$), income tax and NIC as automatic stabilisers ($S^t$) and benefits as automatic stabilisers ($S^b$):

$$\begin{aligned} E &= I[y_k] - I[y] \\ K &= I[y'] - I[y_k] \\ S^t &= I[t(y)] - I[t(y')] \\ S^b &= I[b(t, y')] - I[b(t, y)] \end{aligned} \tag{4}$$

$E$ captures the loss in earnings before the provision of subsidies for furloughed workers ($K$). The stabilisation response of income tax and NIC ($S^t$) is the difference in tax liabilities and NIC based on pre- versus post-crisis pre-tax gross market incomes. The stabilisation response of benefits ($S^b$) is then the difference in benefit entitlements, prior to the benefit increases, based on post- versus pre-crisis after-tax market incomes.

If $I$ is not additively decomposable by income source, e.g. an inequality indicator such as the Gini coefficient, we approximate the contribution of automatic stabilisers by taking the difference in $I$ based on different income concepts:

$$\begin{gathered} E+K = I[y']-I[y] \\ S^t = I[y'-t(y')]-I[y-t(y)]-(E+K) \\ S^b = I[y'-t(y')+b(t,y')]-I[y-t(y)+b(t,y)]-(I[y'-t(y')]-I[y-t(y)]) = \\ = I[C]-I[B]-S^t-(E+K) \end{gathered} \tag{5}$$

where $I[y'-t(y')]/I[y-t(y)]$ is estimated based on gross market incomes net of taxes and NIC. Thus, $E+K$ captures the change in $I$ due to earnings losses net of subsidies; the stabilisation response of income tax and NIC ($S^t$) equals the change in $I$ based on after-tax market incomes, net of $E+K$; and the stabilisation response of benefits ($S^b$) equals the change in $I$ based on household net incomes, prior to the benefit increases, minus $S^t$ and $E+K$.[4]

## 3.2 The data and simulation of shocks

### 3.2.1 The FRS and UKHLS COVID-19 study

Actual data based on household surveys on all components of household net incomes during the pandemic will only be available with a few years' lag. To predict household incomes during the pandemic, we therefore combine the Family Resources Survey (FRS) micro-data for 2018/19 (DWP 2020) with information on employment and earnings shocks from the Understanding Society (UK Household Longitudinal Study-UKHLS) COVID19 Study (University of Essex 2020a). The UKHLS COVID-19 Study did not collect information on all components of income and so cannot be directly used for distributional analysis of household net incomes during the pandemic. Our approach is thus to estimate models of (self-)employment on the UKHLS COVID-19 data, restricted to those with positive earnings prior to the pandemic, and then apply the estimated coefficients from those models on the FRS sample of workers to predict their labour market status in April and May 2020.

The FRS data are the official data source for income statistics in the UK used by the Department for Work and Pensions and are used by the Institute for Fiscal Studies in their annual reports on income trends. We use the latest FRS data for 2018/19, available at the time of writing. We uprate the financial values in the FRS income data to (pre-Covid-19) 2020 prices to account for growth in average earnings and statutory indexation for disability benefits, public pensions, statutory maternity pay, maternity allowance and the statutory sickness pay (we do not directly simulate changes in the labour market and population structure since 2018–19). These are the data used in the *baseline*, i.e. before the employment and earnings shocks, and so they are our simulation of what the UK population would have looked like in April and May 2020 had there been no coronavirus crisis.

The UKHLS is a long-running annual household panel study, and the UKHLS COVID-19 data, which were collected through an on-line and phone survey in April and May 2020, were an addition to the usual annual waves that collected information on the labour market situation

[4] We can rewrite the effect of earnings changes and automatic stabilisers as: $I[y'-t(y')+b(t,y')]-I[y-t(y)+b(t,y)] = I[y']-I[t(y')]+I[b(t,y')]-(I[y]-I[t(y)]+I[b(t,y)])+\eta$, where $\eta$ is a residual term. For additively decomposable measures, such as mean income, $\eta$ is zero but it is non-zero for measures which are not additively decomposable by income source, such as Gini. Hence, when we decompose the change in inequality indicators, our estimate for the contribution of automatic stabilisers contains a residual.

and earnings of individuals in April and May 2020 and how these had changed since February 2020 (i.e. before the crisis had affected the labour market in the UK). The data are of high quality because they are based on reliable probability samples and include carefully constructed and validated weights.[5] The next subsection explains in detail the simulation of the labour market shocks.

### 3.2.2 The simulation of shocks

To reflect the labour market changes seen in the UKHLS COVID-19 data in the FRS, our approach is to estimate a set of equations predicting job loss or furlough on the UKLHS data, and then to use those estimated equations to simulate the same sort of changes in the FRS (in scenarios *C* and *D*).

We estimate two multinomial logit models on the UKHLS COVID-19 data, separately for the April and May waves, one on a sample of employed adults and one on a sample of self-employed adults, both restricted to those who had positive earnings in February 2020 (before Covid-19). For the sample of formerly employed workers, the dependent variable is employment status in April/May 2020 and has four outcomes: i) still employed and with no drop in earnings, ii) still employed (not furloughed) but with reduced working hours and earnings, iii) furloughed, and iv) out of work.[6] The control variables include sex, age and industry (13 categories) and their interactions; household type; baseline earnings quintile; and number of baseline working hours in bands by sex. For the sample of formerly self-employed workers, the dependent variable is self-employment status in April/May 2020 with the following three outcomes: a) still self-employed and with no drop in earnings, b) still self-employed but with reduced hours and earnings, and c) out of work. The control variables include sex, age, industry, household type, baseline earnings ventile, and number of baseline working hours in bands by sex. In addition, the May wave of the UKHLS COVID-19 data includes information on entitlement to the SEISS grant. We run a logit model on the sample of formerly self-employed workers to estimate the likelihood of receiving the SEISS grant, controlling for the change in self-employment status since the coronavirus crisis. Results from the multinomial logit, based on the April and May waves separately, are reported in Table A.1 (for the employed) and Table A.2 (for the self-employed), and results from the logit model, based on the May wave only, are reported in Table A.3.[7]

We use the estimated coefficients from the multinomial logit to predict the probability of each outcome for each individual in the FRS sample of workers with positive earnings. We then draw from a uniform distribution a random number, equal to the labour market outcome

[5] Crossley et al. (2021) describe the weighting strategy in the UKHLS COVID-19 Study and carefully validate the weights on an extensive set of variables, applying statistical tests of the weighting effectiveness. They find that the weights perform very well and can replicate accurately population totals on a wide range of characteristics such as subjective finances, type of housing tenure, being poor or having a long-standing illness. In particular, the data are of high quality for two key reasons: First, the data are derived from reliable probability samples. This means that every unit in the target population has a knowable, non-zero probability of selection and so, with large enough samples, the full heterogeneity of the target population will be captured. Second, the set of cross-sectional weights, which we use in this paper, are constructed using a set of very detailed individual characteristics on both respondents and non-respondents (e.g. basic demographics, household composition, economic and health information and information on types of contact).

[6] *No drop in earnings* constitutes a drop of no more than £5 per week. *Reduced hours and earnings* implies a fall of more than £5 in earnings and at least 1 working hour per week.

[7] Industry was collected at Wave 9 (2017–18) of Understanding Society (University of Essex 2020b).

of the worker, where each possible outcome has a probability equal to the predicted probability. Following the same approach, we use the estimated coefficients from the logit model and the simulated self-employment status after the shock to predict entitlement to the SEISS grant. In total, 1.1 million workers in the FRS are simulated to be out of work (having applied the grossing weights), 7.3 million workers to be furloughed and 3.1 million workers to experience a drop in working hours and earnings (but not furloughed). Thus, the majority of workers affected by the initial Covid-19 shock experienced a drop in earnings rather than a complete loss in earned income. Overall, 1.8 million self-employed are simulated to receive a grant from the SEISS. See Appendix C for further details on the simulation of the CJRS subsidy and SEISS grant.

Finally, employed and self-employed in the FRS with hours and earnings reduction (outcomes ii and b, respectively) experience a drop in earnings and hours which we simulate as the mean relative drop in earnings by sex in the UKHLS COVID-19 sample amongst those reporting a drop. For employed, the drop is 41% for men and 43% for women in April and 21% for both men and women in May. For self-employed, the drop is 74% for both men and women in April and 62% for men and 56% for women in May.

Table 1 shows the characteristics of workers in the FRS by employment status after the simulated shocks. There is a clear age, education and income gradient among the workers affected by the shocks, matching what was found in Crossley et al. (2021) who used the same UKHLS COVID-19 data. A larger share of those out of work are aged 20–29 compared to the other age groups. Those out of work are also more likely to come from the lowest earnings quintile. Furloughed workers are more likely to be young, male, lower educated, and to be from the bottom and middle of the earnings distribution. In comparison, those with reduced working hours and earnings (but not furloughed) are more likely to be older, male, and in the bottom earnings quintile. Those with no drop in their earnings (‘no change’) are more likely to be older, female, higher educated, and from the upper part of the earnings distribution.

Tables 2 and 3 show the distribution of workers in the FRS by industry and occupation, respectively, by employment status after the simulated shocks. Workers in accommodation and food services, construction and real estate, and the arts, entertainment and recreation are most likely to become out of work, furloughed or have their earnings and hours reduced, as are workers in elementary, skilled trades and process, plant and machine operatives occupations.

### 3.3 The tax-benefit model UKMOD

The measure of household net income in this paper is cash income and is the sum of gross market incomes, state pensions, national insurance and means-tested benefits minus income tax liabilities and NIC, equivalised using the modified OECD equivalence scale rescaled to 1 for a couple without children (with a value of 0.67 for the first adult aged 14+, 0.33 for any other adult and 0.2 for any child aged <14).

To calculate income tax liabilities, NIC and entitlements to state benefits, we use the tax-benefit model UKMOD, a spin-off model of the UK component in the EU-wide tax-benefit model EUROMOD (see Sutherland and Figari (2013) and Figari et al. (2015) for information on EUROMOD and Reis and Tasseva (2020) for information on UKMOD). UKMOD simulates for each individual and household in the FRS their taxes and benefits based on their gross earnings, other sources of income, their individual and household characteristics, and the tax-benefit measures that were in place at a given time period. UKMOD calculations account for non-take-up of income-related benefits (see Appendix D).

**Table 1** Characteristics of workers by simulated employment status

| | Out of work | Furloughed | Reduced hours & earnings | No change |
|---|---|---|---|---|
| Age group: | | | | |
| 20–29 | 6.0 | 34.5 | 7.1 | 52.4 |
| 30–39 | 3.9 | 25.6 | 10.7 | 59.7 |
| 40–49 | 3.5 | 23.4 | 11.9 | 61.3 |
| 50–59 | 2.7 | 22.2 | 13.5 | 61.6 |
| 60+ | 3.0 | 24.6 | 16.4 | 56.1 |
| Men | 4.0 | 27.7 | 12.1 | 56.2 |
| Women | 3.9 | 24.5 | 10.2 | 61.5 |
| In a household with: | | | | |
| children | 4.8 | 26.7 | 10.4 | 58.1 |
| 1 earner | 3.4 | 25.4 | 11.8 | 59.4 |
| 2+ earners | 4.1 | 26.5 | 11.0 | 58.5 |
| Completed education aged: | | | | |
| 16 or less | 3.8 | 29.6 | 12.0 | 54.6 |
| 17–19 | 3.9 | 28.6 | 10.6 | 56.9 |
| 20+ | 4.1 | 20.9 | 11.0 | 64.0 |
| Earnings quintile: | | | | |
| 1 | 8.5 | 31.8 | 15.2 | 44.5 |
| 2 | 2.9 | 33.5 | 11.9 | 51.7 |
| 3 | 2.7 | 29.2 | 9.9 | 58.3 |
| 4 | 3.6 | 22.2 | 8.7 | 65.5 |
| 5 | 2.5 | 15.4 | 10.8 | 71.4 |
| Number of (in thousand): | | | | |
| employed | 956 | 7291 | 1343 | 14,621 |
| self-employed | 140 | 0 | 1769 | 1722 |
| all | 1096 | 7291 | 3112 | 16,343 |
| Receiving SEISS grant | 114 | 0 | 1185 | 491 |

The table shows the share of workers (in %) by different characteristics simulated to be out of work, furloughed, with reduced hours and earnings (not furloughed) and with no drop to earnings (no change). The results are based on average monthly estimates, except for the number of those receiving the SEISS grant which refers to May only. The sample includes individuals with positive earnings from employment and/or self-employment and aged 20–63. Employed with earnings from employment only. Self-employed with earnings from self-employment (and employment)

*Source:* Own calculations with FRS and Understanding Society COVID-19 data

In the baseline and scenario *C*, UKMOD calculates net incomes based on the tax-benefit rules that would have applied in April/May 2020 in a world where the Covid-19 did not happen, i.e. using the policies that were confirmed in the government Budget on 11 March 2020. The amount of income tax and NIC paid and state benefits received by households differ between the baseline and scenario *C* because of the simulated shocks in *C*. By comparing the baseline and scenario *C*, we can estimate the fiscal and distributional impact of the earnings subsidies and assess the effectiveness of the pre-Covid tax-benefit system in cushioning economic shocks. We refer to this as the automatic stabilisation effect of policies (see Paulus and Tasseva 2020; Dolls et al. 2012).

In scenario *D*, UKMOD re-calculates net incomes accounting for the benefit increases that the UK government introduced in response to the crisis. Thus, although the earnings data are the same in scenarios *C* and *D*, the amount of state benefits received by households differ because scenario *D* includes the Covid-related increases in benefit levels (there are no changes in taxes paid by households between scenarios *C* and *D*).

**Table 2** Share of workers (in %) by industry

| | Out of work | Furloughed | Reduced hours & earnings | No change |
|---|---|---|---|---|
| Agriculture, Mining, Manufacture, Utilities | 5.1 | 36.9 | 7.9 | 50.1 |
| Construction and Real Estate | 2.4 | 38.4 | 19.8 | 39.4 |
| Wholesale and Retail Trade | 5.6 | 36.1 | 6.8 | 51.5 |
| Transportation and Storage | 1.8 | 35.1 | 13.2 | 49.9 |
| Accommodation and Food | 11.5 | 57.2 | 10.2 | 21.1 |
| Information and Communication | 3.1 | 8.5 | 10.7 | 77.8 |
| Financial and Insurance Activities | 2.2 | 6.2 | 4.0 | 87.7 |
| Professional, Scientific and Technical Activities | 1.8 | 18.4 | 14.8 | 65.0 |
| Administrative and Support Service | 2.3 | 31.1 | 21.6 | 45.1 |
| Public Administration and Defence | 1.8 | 8.2 | 4.6 | 85.4 |
| Education | 4.0 | 13.7 | 10.6 | 71.7 |
| Human Health and Social Work | 4.5 | 12.1 | 9.0 | 74.4 |
| Arts, Entertainment, Recreation and Other | 2.0 | 37.3 | 18.8 | 42.0 |

The table shows, for a given sector, the share of workers (in %) simulated to be out of work, furloughed, with reduced hours and earnings (not furloughed) and with no drop to earnings (no change). The results are based on average monthly estimates. The sample includes individuals with positive earnings from employment and/or self-employment and aged 20–63

*Source:* Own calculations with the FRS and Understanding Society COVID-19 data

UKMOD calculations for scenarios *C* and *D* are done separately for April and May. In the remainder of the paper, we show results for the income distribution in scenarios *C* and *D*, based on household incomes averaged over April and May.

We focus on the distributional impact of policies, holding everything else constant and thereby, abstracting from any macroeconomic adjustments and behavioural reactions to the policies we simulate. These adjustments could have taken a number of forms. In one thought experiment, if there had been no fiscal response to the crisis – i.e. the government had not introduced the CJRS, SEISS and increases to UC and other benefits – then there would have been a much larger fall in household incomes than we simulate, and this would have led to further drops in incomes and demand through the multiplier process (Christiano et al. 2011; Auerbach and Gorodnichenko 2012). Working in the other direction is the possibility that the existence of the CJRS programme could have led some employers to furlough some workers who would otherwise have remained employed and on full-pay (in other words, the actual counterfactual to the CJRS being introduced is not necessarily one where all furloughed workers are instead made redundant). Additionally, it is possible that the increased UC entitlements and high media coverage of the government's emergency package could have led to higher take-up of UC (Currie 2006) by lowering transaction costs and information barriers. In our analysis, we keep benefit take-up constant (see Appendix D) and thus, in the presence of take-up increases we provide a lower bound estimate for the impact of UC on incomes. Finally, the introduction of SEISS and the increased entitlements to UC could have led to reductions in labour supply through the income effect, although we expect these to be small overall due to the severe labour demand constraints and economic uncertainty in the period of analysis.

**Table 3** Share of workers (in %) by occupation

| | Out of work | Furloughed | Reduced hours & earnings | No change |
|---|---|---|---|---|
| Missing | .0 | 17.9 | 6.1 | 76.0 |
| Managers, Directors and Senior Officials | 3.0 | 24.6 | 12.3 | 60.2 |
| Professional Occupations | 3.0 | 15.3 | 9.7 | 72.1 |
| Associate Prof. and Technical Occupations | 2.7 | 18.8 | 11.0 | 67.5 |
| Admin and Secretarial Occupations | 3.3 | 26.4 | 7.4 | 62.9 |
| Skilled Trades Occupations | 4.1 | 32.8 | 19.5 | 43.6 |
| Caring, Leisure and Other Service Occupations | 4.9 | 21.3 | 11.2 | 62.6 |
| Sales and Customer Service | 7.0 | 36.0 | 6.0 | 51.0 |
| Process, Plant and Machine Operatives | 4.2 | 35.5 | 13.5 | 46.8 |
| Elementary Occupations | 5.9 | 44.7 | 10.1 | 39.2 |

The table shows, for a given occupation, the share of workers (in %) simulated to be out of work, furloughed, with reduced hours and earnings (not furloughed) and with no drop to earnings (no change). The results are based on average monthly estimates. The sample includes individuals with positive earnings from employment and/or self-employment and aged 20–63

*Source:* Own calculations with the FRS and Understanding Society COVID-19 data

## 4 Results

This section assesses the impact of the crisis, and of the UK policy response to Covid-19, on the fiscal budget and the household income distribution. It also looks in more detail at the impact of the increase to state benefits announced by the government when the crisis hit, as well as at how effective the tax-benefit system would have been in responding to the economic shocks without these measures. It then analyses the impact of a near-decade austerity on the UK safety net and the distributional impact of replacing the emergency policies with a budget-neutral Universal Basic Income.

### 4.1 Changes to earnings and the fiscal budget

Table 4 reports the level and changes to total employee and self-employed monthly earnings, subsidies from CJRS and SEISS, government revenues from income tax and NIC, and government spending on state benefits. Column 2 shows the levels in the baseline (before Covid-19). Column 3 shows the impact of the Covid employment and earnings shocks and the full UK policy response (i.e. scenario *D* versus the baseline) in million £. Table 5 shows the baseline number and the change to the number of earners, taxpayers and state benefit recipients.

#### 4.1.1 Earnings and earnings subsidies from the CJRS and SEISS

The labour market shock means that total employee and self-employed earnings fell by £8 billion (11.4%) and £3.2 billion (33.4%), respectively. This was mainly due to workers and self-employed experiencing a cut to their earnings rather than losing their job. Subsidies from the CJRS amounted to a substantial £10.7 billion, or 15.3% of total employee earnings in the baseline. Spending on grants from the SEISS amounted to £2.1 billion, or 22% of total baseline self-employed earnings. The government is simulated to have received about 20.1% of the spending on CJRS and SEISS back in the form of income tax and NIC.

The shock is simulated to have reduced the number of employed and self-employed workers by 956,000 and 140,000, respectively, while 7.3 million workers are simulated to have been furloughed and on the CJRS and 1.8 million self-employed to have received an SEISS grant. This compares to an estimated 8.4 million jobs that had been furloughed and 2.4 million claims to the SEISS by 31 May.[8] In our simulations, we move in total 1.1 million workers out of work.[9]

### 4.1.2 Tax revenues and benefit spending

As earnings fell, revenues from income tax also fell by a substantial £2.3 billion (16.2%) per month, and revenues from NIC by £1 billion (13%). This translates to 1.5 million people being fully exempt from paying income tax and NIC as their income fell below the Personal Allowance and the NIC Primary Threshold.

Benefit spending went up substantially. This was mainly due to the spending expansion of the main means-tested benefit, UC, as well as the contributory unemployment benefit, JSA. There was also increased spending on other benefits (WTC, HB, CTR and CB).

Spending on UC expanded substantially by £0.8 billion (28.2%) and the number of UC claimants are simulated to have risen by 1.1 million (27.2%) to 5.2 million. Thus, UC provided important safety net during this crisis. For comparison, based on figures published by the Department for Work and Pensions, the record number of claims led to 2.4 million starts to UC in the period 13 March 2020 to 14 May 2020 bringing the total number of people on UC to 5.3 million.[10] The savings made due to the benefit cap applied on UC were noteworthy, and are simulated to have been worth £334 per affected family per month, on average (although they made up a small proportion of the total spend on UC).

Spending on JSA also increased substantially by £261 million, and the number of JSA claims is simulated to have risen by 837,000 due to the sharp rise in the number of unemployed, highlighting the effectiveness of unemployment benefits in providing insurance during economic downturn (Dolls et al. 2012; Auerbach and Feenberg 2000).

## 4.2 The impact of the crisis on the distribution of income

### 4.2.1 The distributional impact of the shock to earnings, and of the tax-benefit response

Figure 1 shows the distributional impact of the shock to earnings and the tax-benefit response by showing the difference in mean equivalised household net income between the baseline and scenario *D*, where households are ranked based on their net income before Covid-19. As well as showing the change in net income (black circle), we also show the change due to employee earnings and self-employed earnings; subsidies from CJRS (people simulated to be put on the

[8] HM Revenue & Customs, Coronavirus Job Retention Scheme statistics: December 2020 and Self-Employment Income Support Scheme statistics: June 2020, available at https://www.gov.uk/government/collections/hmrc-coronavirus-covid-19-statistics.

[9] According to HM Revenue and Customs' Pay As You Earn Real Time Information from July 2020 the number of paid employees has fallen by 585,530 by May 2020 compared to February 2020, while Office for National Statistics data from January 2021 estimate that the number of self-employed has fallen by 248,000 in April–June 2020 compared to January–March 2020 (both datasets available at https://www.ons.gov.uk/employmentandlabourmarket).

[10] https://www.gov.uk/government/statistics/universal-credit-statistics-29-april-2013-to-9-july-2020/universal-credit-statistics-29-april-2013-to-9-july-2020#starts-on-uc-header

**Table 4** Change in total earnings, revenues and spending (in million £ and per month)

| | Baseline (In levels) | Impact of crisis (Change to baseline) |
|---|---|---|
| Employee earnings | 69,906 | −7961 |
| Self-employed earnings | 9593 | −3208 |
| Earnings subsidies from CJRS | 0 | 10,690 |
| Earnings subsidies from SEISS* | 0 | 2114 |
| Revenues from income tax+NIC | 22,005 | −3317 |
| Income tax | 14,308 | −2317 |
| Employee NIC | 7266 | −891 |
| Self-employed NIC | 432 | −109 |
| Spending on benefits | 6163 | 1206 |
| Universal Credit (UC) | 2860 | 805 |
| *Savings from UC benefit cap* | 27 | 14 |
| Jobseeker's Allowance (JSA) | 10 | 261 |
| Working Tax Credit (WTC) | 76 | 28 |
| Housing Benefit (HB) | 707 | 24 |
| Child Benefit (CB) | 961 | 26 |
| Council Tax Reduction (CTR) | 385 | 26 |
| Other means-tested benefits | 1164 | 36 |
| Revenues – spending | 15,842 | −16,270 |

*Spending on SEISS for May only. All other results are based on average monthly estimates. Other means-tested benefits include the Child Tax Credit, Income support, income-related Employment and Support Allowance, income-based JSA, Pension Credit, Scottish benefits (Sure Start Maternity Grant and Best Start Grant). No simulations to Statutory Sickness Pay

*Source:* Own calculations with UKMOD and FRS

**Table 5** Change in number of earners, tax payers and state benefit recipients (in thousand)

| | Baseline (In levels) | Impact of crisis (Change to baseline) |
|---|---|---|
| Employed | 26,714 | −956 |
| Self-employed | 4167 | −140 |
| Furloughed workers on CJRS | 0 | 7291 |
| Self-employed on SEISS* | 0 | 1791 |
| Income tax+NIC payers | 32,769 | −1497 |
| Income tax | 29,925 | −1977 |
| Employee NIC | 23,908 | −1047 |
| Self-employed NIC | 3057 | −531 |
| Benefit recipients | 13,313 | 1471 |
| Universal Credit (UC) | 4071 | 1107 |
| *Affected by UC benefit cap* | 82 | 40 |
| Jobseeker's Allowance (JSA) | 34 | 837 |
| Working Tax Credit (WTC) | 298 | 0 |
| Housing Benefit (HB) | 2001 | 8 |
| Child Benefit (CB) | 7107 | 159 |
| Council Tax Reduction (CTR) | 5270 | 322 |
| Other means-tested benefits | 3357 | 57 |

*Number of self-employed on SEISS in May 2020. All other results are based on average monthly estimates. Other means-tested benefits include the Child Tax Credit, Income support, income-related Employment and Support Allowance, income-based JSA, Pension Credit, Scottish benefits (Sure Start Maternity Grant and Best Start Grant). No simulations to Statutory Sickness Pay

*Source:* Own calculations with UKMOD and FRS

CJRS are treated as if they lost all their earnings but gained a new source of income: the CJRS payment) and SEISS; income tax + NIC; and all benefits (see eqs. 3 and 4 in Section 3.1). A negative change means a fall in net income.

In more detail, we estimate that household net income fell by 6.9% on average. Breaking down the change by income source shows that earnings subsidies from the CJRS accounted for a large share of the average baseline income, i.e. 13.2%. Subsidies from the SEISS contributed to a smaller income gain of 1.3% on average. Thus, *after* accounting for the CJRS and SEISS grant, the loss to earnings from both employment and self-employment amounted to 12.6% of baseline net income. Automatic reductions in income taxes and NIC also helped mitigate income losses, contributing to an average gain in net income of 4.1%. A smaller gain of 1.6% came from state benefits.

Across the income distribution, the top eight decile groups lost out, on average, with losses proportionally larger in the middle and top of the distribution than at the bottom. The fact that the losses in earned income were skewed towards high-income families mostly reflects the way earners are distributed across the household income distribution: there are substantially more no-earner and fewer two-earner households at the bottom than in the middle or top of the distribution (Table A.4). If we only focus on households with one earner, then the losses in earned income, as a proportion of baseline net income, were largest at the bottom of the distribution (Fig. B.1). The losses in earned income for households with two-and-more earners were somewhat more equally distributed (Fig. B.2).[11]

Subsidies from CJRS played a crucial role in supporting household finances across the entire distribution. They provided the main insurance mechanism during the crisis. Subsidies from SEISS also provided income support across the distribution, with somewhat larger benefits at the bottom. The CJRS and SEISS payments combined provided net income gains amounting to 10.5% of the baseline net income in the poorest decile up to 19.5% in the seventh decile. In the richest decile, the cap on the CJRS subsidy made the scheme less effective in insuring household incomes against the shocks.

After the earnings subsidies, reductions in income tax and NIC also helped mitigate income losses. They absorbed a higher proportion of the losses the higher the decile group, due to the progressive nature of the income tax and NIC schedule. In other words, income tax and NIC shielded higher-earners more than lower-earners.

At the bottom of the distribution, state benefits played an important role in alleviating pressure on the households budgets. In the bottom three deciles, benefits contributed to sizeable income gains of 4–5%.[12]

In addition, Fig. 1 shows the change in net income had there been no emergency measures introduced (hollow diamond), i.e. no CJRS and SEISS and no increases to UC and other means-tested benefits. In this scenario with no emergency measures, we assume at the extreme that all furloughed workers would have been made unemployed in the absence of the CJRS

[11] These results are broadly consistent with the estimates for the distributional impact of the pandemic on working households by HM Treasury (2020).

[12] Figure B.3 shows changes in net income by industry, for the sample of workers with positive earnings in the baseline (before Covid-19). Although the hits to household earnings varied considerably by industry the overall change in worker's household net income by industry varied less so. Broadly, the change in net income ranged from −4.5% in "Financial and Insurance Activities" and "Public Administration and Defence" to −9.2% up to −10.3% in "Accommodation and Food" and "Construction and Real Estate", respectively. The key finding remains that earnings subsidies from the CJRS provided the main mechanism for insurance against the shocks and income smoothing; the SEISS grant and tax-benefit policies also provided important income protection.

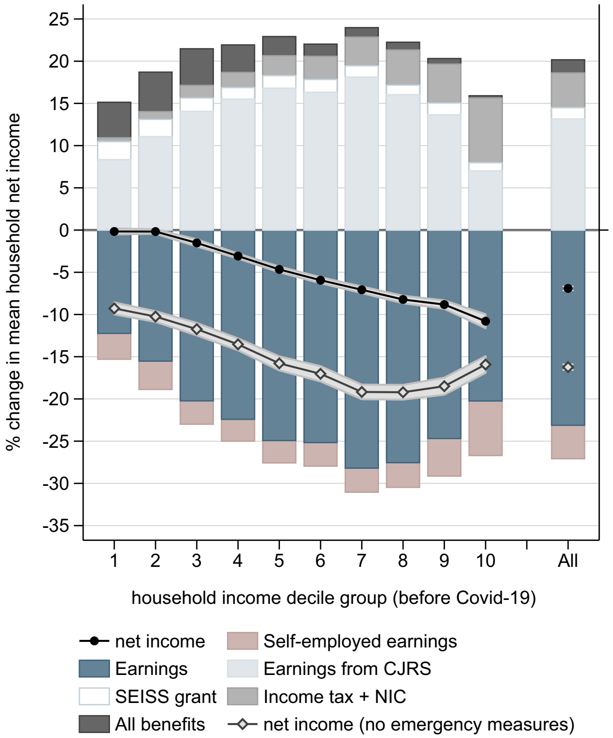

**Fig. 1** Impact of the crisis and policy response to Covid-19 on mean net income by decile of *pre-crisis* income. *Notes:* The figure shows the distributional impact of the employment and earnings shocks and the UK policy response, i.e. the *baseline* versus scenario *D*. Changes in net income are broken down by income source. Additional results for the change in net income in the absence of the Covid emergency measures (i.e. CJRS, SEISS and increases to UC and other means-tested benefits) are also shown. Results based on average monthly estimates. Changes in income based on equivalised household net income. *Source:* Own calculations using UKMOD and FRS

and would have received instead the unemployment benefit JSA and the means-tested benefit UC. Keeping this in mind, the difference between the two net income scenarios – with and without emergency measures – can be thus thought of as the effect of the government emergency policies. We find that without the emergency measures household net income would have fallen by 16.2% on average, i.e. a substantial additional income loss of 9.3 pp. compared to the scenario with the emergency policies. Across the distribution, the loss in net income would have been between 9.1 pp. and 12.1 pp. higher for deciles 1 to 9 and 5.1 pp. higher for decile 10 in the absence of the emergency measures. To sum up, we show that the emergency measures introduced in response to the pandemic mitigated substantially the loss to household incomes.

### 4.2.2 Changes in the distribution of income

Figure 2 presents the change in net income in a different way, by comparing the level and composition of household net incomes in the baseline and scenario *D*, and doing this within each decile group having re-ranked households. If income changes are relatively small, then

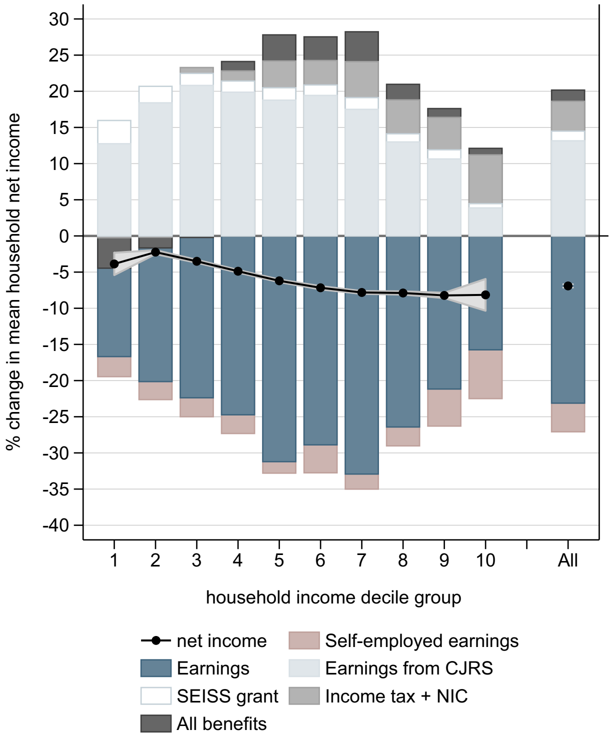

**Fig. 2** Impact of the crisis and policy response to Covid-19 on mean net income; households ranking not fixed. *Notes:* The figure shows the distributional impact of the employment and earnings shocks and the UK policy response, i.e. the *baseline* versus scenario *D*. Results based on average monthly estimates. Changes in income based on equivalised household net income. Households ranking based on the respective (baseline or post-Covid) income distribution. *Source:* Own calculations using UKMOD and FRS

individuals will retain their initial (before the crisis) relative position in the income distribution (and so, Figs. 1 and 2 will show two very similar pictures). But if income changes are large, then some individuals experiencing the negative impact of the initial Covid-19 shock may be pushed down the distribution. Indeed, we find that the pattern of income changes is somewhat different from that in Fig. 1 showing that this re-ranking effect is large. Figure 2 illustrates that the crisis is simulated to have led to a large rise in the number of adults with very low disposable income (caused by those who lost all their earnings as a result of the crisis), such that average incomes in the bottom decile were 3.9% lower.

Figure 3 shows the impact of Covid-19 on income inequality based on the Gini coefficient, coefficient of variation (CV), mean log deviation (MLD) and the Theil index (TI). The total change in inequality (black dot) is decomposed into the contribution of earnings changes (i.e. earnings losses net of subsidies) and of tax-benefit policies (taken all together). In line with Fig. 2, we find that net income inequality fell slightly, with the drop being statistically significantly different from zero for all indices apart from the CV. Disparities in earnings increased although the increase is statistically significant only for the Gini coefficient (0.005). However, tax-benefit policies together more than offset the increased inequality in earned income. We return to the distributional impact separately of income tax+NIC and of state benefits in the next section.

**Fig. 3** Impact of the crisis and policy response to Covid-19 on income inequality. *Notes:* The figure shows the impact on inequality of the employment and earnings shocks and the UK policy response, i.e. the *baseline* versus scenario *D*. CV = coefficient of variation; MLD = mean log deviation; TI = Theil index. Results based on average monthly estimates of income. Changes in income inequality based on equivalised household net income. *Source:* Own calculations using UKMOD and FRS

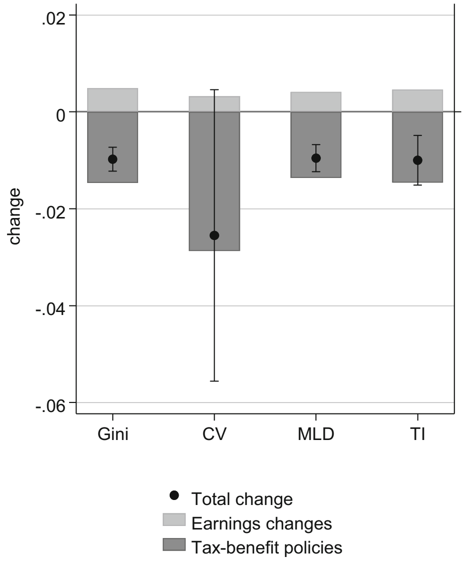

We also look at changes in income poverty, measured against a fixed and a floating poverty line. The two measures used together provide a fuller picture of poverty and inequality at the bottom of the income distribution (and both are currently monitored by the UK government). With a fixed poverty line we capture changes in the real living standards of individuals. With a floating poverty line, what we capture is closer to a change in inequality at the bottom of the income distribution. We estimate that the poverty rate and poverty gap (Table 6 and Table A.5, respectively) increased due to Covid-19 using a fixed poverty line at 60% of the median baseline income, illustrating a drop in living standards due to the crisis. But the poverty rate and gap fell using a floating poverty line due to a significant drop in median income and a compression of incomes at the bottom of the distribution. For the overall population, the poverty rate increased from 16.5% to 17.7% with a fixed poverty line while it went down to 13.7% with a floating poverty line.

### 4.3 The distributional impact of the Covid-19 increases to state benefits and tax-benefit stabilisers

In this section, we analyse separately the contribution of the state benefit increases announced in response to Covid-19 and the contribution of the tax-benefit system that we would have had in their absence (i.e. the one confirmed in the government's March 2020 Budget): we refer to this latter one as the automatic stabilisers.

Figure 4 repeats the change in net income due to state benefit entitlements, and income tax and NIC liabilities that was shown in Fig. 1, but now shows in the left plot, the impact of the automatic stabilisers and in the right plot, the impact of Covid-related benefit increases (for the estimation of the effect of benefit increases and automatic stabilisers, see Eqs. 3 and 4, respectively, in Section 3.1). Comparing the total impact of the automatic stabilisers versus the total effect of Covid-related benefit increases on mean net incomes, it is clear that the former had the most impact on net incomes, on average. This underlines the importance of tax-benefit designs in protecting household incomes during economic downturn. On the other

**Table 6** Change in the poverty rate in % points

| | Baseline (in %) | Impact of crisis (Change to baseline) | |
|---|---|---|---|
| | | Fixed | Floating |
| All | 16.491*** | 1.173*** | −2.744*** |
| | (.350) | (.226) | (.257) |
| Women | 17.040*** | 1.161*** | −2.873*** |
| | (.397) | (.215) | (.268) |
| Men | 15.927*** | 1.185*** | −2.612*** |
| | (.388) | (.268) | (.293) |
| Children | 21.505*** | 1.514*** | −4.484*** |
| | (.715) | (.461) | (.491) |

The table shows the impact on the poverty rate of the employment and earnings shocks and the UK policy response, i.e. the *baseline* versus scenario *D*. The poverty rate is the % of people with household equivalised net income below the poverty line. The poverty line is 60% of the median household equivalised net income in the baseline (fixed) or of the respective scenario (floating). Standard errors at a confidence level of 95% are shown in parenthesis. Bootstrapped standard errors after 200 replications. Significance levels indicated as * $p < 0.1$, ** $p < 0.05$, *** $p < 0.01$

*Source:* Own calculations with UKMOD and FRS

hand, different parts of the distribution rely on different types of policy for mitigating income shocks, stressing the importance of both automatic stabilisers and governments' responses to crises for providing income protection (see e.g. Paulus and Tasseva 2020).

In more detail, looking at the plot on the left, income taxes and NIC had the most sizeable effect on mean net income. Furthermore, they compensated most for income losses at the upper end of the distribution, due to the progressivity of the system. In the richest top decile, income tax and NIC contributed to a gain of 6.3% and 1.3%, respectively. In other words, after the drop in earnings, the top decile retained in total 7.7% of its baseline income due to lower tax liabilities and NIC. This emphasises the role of income taxes and NIC as an important source for income insurance (Kniesner and Ziliak 2002a, 2002b).

However, income taxes and NIC had less impact at the bottom of the distribution where, on average, they accounted for a much smaller share of baseline income (for example, someone earning £16,800 per year, i.e. working 37 h a week at the National Living Wage level, would pay 5% of gross income in income tax, compared to 15% if earning £50,000 per year).

What protects most households at the bottom of the distribution during recessions are state benefits: in the case of the current crisis it was mainly the means-tested benefit UC. As a stabiliser (left plot), UC provided a safety-net mainly for the bottom six deciles. The Covid-related increases to UC (right plot) contributed to additional income gains targeted at the bottom of the distribution. For the poorest decile, this amounted to an average gain in equivalised net income of 2.8%. The stabilisation effect of UC and the UC emergency increase benefited most households with children, lone mothers, one-earner families and those in privately rented or social housing (Fig. B.7). After UC, the unemployment benefit JSA (left plot) also contributed to small income gains across all parts of the distribution highlighting the importance of provision and access to social insurance benefits. For households with two-and-more earners the gains from JSA were quite sizeable, especially in the bottom five deciles (Fig. B.5). The increases to WTC and HB (right plot) had a small positive effect on net incomes.

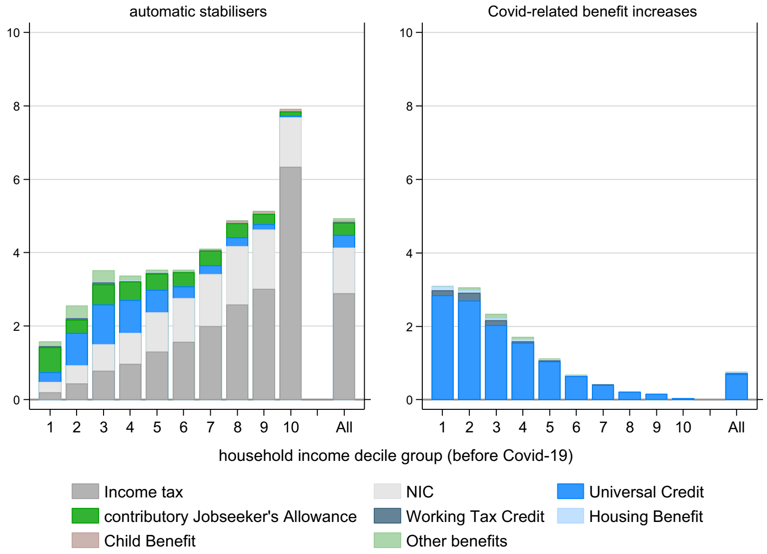

**Fig. 4** Impact of the crisis and policy response to Covid-19 on mean net income by decile and tax-benefit policy. *Notes:* Left plot shows impact of automatic stabilisers, i.e. the *baseline* versus scenario *C*. Right plot shows impact of Covid-related benefit increases, i.e. scenario *C* versus *D*. Results based on average monthly estimates. Changes in total net income and the contribution of earnings changes, CJRS subsidies and SEISS grant are omitted. Changes in income based on equivalised household net income. Other benefits include the Council Tax Reduction, Child Tax Credit, Income support, income-related Employment and Support Allowance, income-based JSA, Pension Credit, Scottish benefits (Sure Start Maternity Grant and Best Start Grant). No simulations to Statutory Sickness Pay. *Source:* Own calculations using UKMOD and FRS

Finally, we assess the separate contribution of benefits (breaking down their contribution into stabilisers and Covid-related benefit increases) and tax+NIC stabilisers to the total change in inequality measured by Gini, CV, MLD and TI (see Eqs. 3 and 5 in Section 3.1 and Table A.6 for detailed results). The key finding is that benefits (both automatic stabilisers and Covid-related benefit increases) contributed to an inequality reduction, highlighting their importance for redistribution; while income tax and NIC had little impact on inequality. Overall, benefits reduced the Gini coefficient by 0.018, CV by 0.036, MLD by 0.016 and TI by 0.017.

### 4.4 The impact of austerity on the UK safety net

In this section, we explore the impact of a near-decade of austerity on the effectiveness of tax-benefit policies in providing income protection. After nearly a decade of more generous benefits in the 2000s, 2011 marks a turning point to a period of retrenchment. Since 2011, there have been various cuts made to eligibility, or freezes made to the nominal benefit entitlements (De Agostini et al. 2018). A key reform has been the introduction of UC, which has been gradually replacing several means-tested benefits into a single payment. While there are both gainers and losers from the reform, UC is disproportionally reducing the incomes among poorer households due to an overall cut to benefit entitlements compared to the programmes it is replacing (Brewer et al. 2019). On the tax side, the top marginal tax rate was reduced from 50% to 45% and the zero tax band (Personal Allowance) has grown faster than prices, but the higher income tax thresholds and the point at which the Personal Allowance is tapered away have deteriorated in real terms.

To assess the impact of austerity on the UK safety net, we compare the distributional impact of tax and benefit policies had the 2011 system been in place today (indexed by CPI) versus the 2020 *pre-Covid* and the 2020 *post-Covid* systems (Fig. 5). To estimate the impact of the 2011 policies, we use the tax-benefit model UKMOD to apply the indexed 2011 tax-benefit policies on the household micro-data a) before Covid-19 and b) after the simulation of employment and earnings shocks.

Comparing the distributional impact of the 2011 with the 2020 *pre-Covid* system, Fig. 5 shows that for all parts of the distribution the 2020 *pre-Covid* system would have been less effective in providing insurance against shocks than the 2011 system. In particular in the poorest decile, the 2011 tax-benefit policies would have raised net income by an additional 2.1% compared to the 2020 *pre-Covid* policies.

The emergency benefit package in response to the pandemic strengthened the UK safety net and, compared to 2011, the 2020 *post-Covid* system provides more insurance in deciles 1 to 5 and about the same in the rest of the distribution. Nevertheless, although the Covid-related benefit increases provide additional income insurance, at the time of writing these protections are temporary and also do not undo the impact of austerity measures over the years.

### 4.5 The impact of a Universal Basic Income

A Universal Basic Income (UBI) is an unconditional universally-paid benefit, which decouples entitlement from work, or any other condition, and is paid on an individual basis. Using microsimulation methods, Reed and Lansley (2016), Atkinson et al.

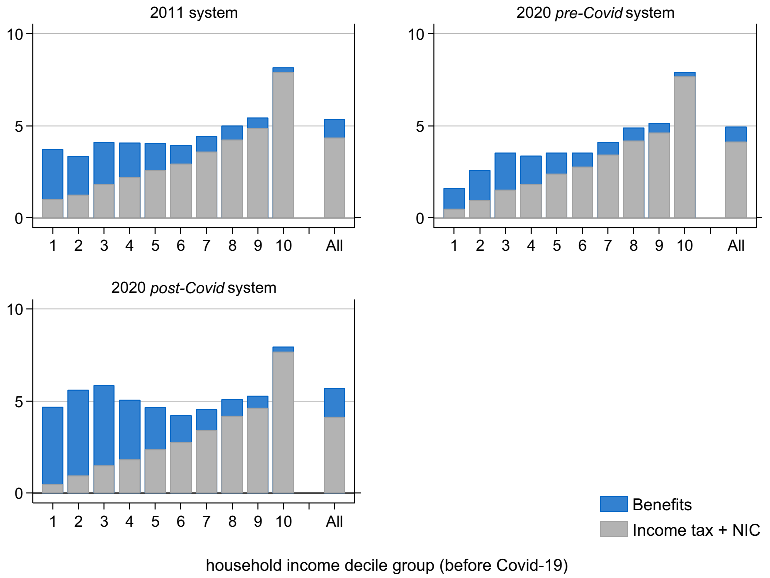

**Fig. 5** The impact of the 2011 versus the 2020 pre−/post-Covid tax-benefit systems on household net incomes *Notes:* 2020 *pre-Covid* and 2020 *post-Covid* systems based on scenarios *C* and *D*, respectively. 2011 tax-benefit parameters in 2020 prices and applied on the earnings distribution before and after Covid-19 to estimate effect of automatic stabilisers. *Source:* Own calculations using UKMOD and FRS

(2017) and Martinelli (2020) assess the potential distributional effect of a UBI in the UK and show that a UBI could lower income poverty and inequality but part of the existing means-tested benefit system needs to be retained to avoid a large number of losers. In March 2020 a cross-party group of parliamentarians called for a UBI to be introduced in response to the pandemic. A UBI, due to its simplicity, would have had two key advantages over the emergency policies that were introduced: it would have put little administrative burden on both government agencies and households to administer and apply for the benefit, respectively, and would have eliminated any gaps in the social security coverage arising from the design of the system and benefit non-take-up.

In this section, we explore what would have been the impact on household incomes if a UBI was introduced in place of the Covid emergency policies (i.e. the CJRS, SEISS and increases to UC and other means-tested benefits). In this hypothetical scenario, we simulate a *budget-neutral* UBI equal to £1744 per year per adult which tops up household incomes, i.e. it is not taxed and does not enter the income-test for any other benefit. Budget neutrality here implies that the fiscal budget (tax revenues – benefit spending) under the UBI scenario is the same as with the emergency policies.[13] In the UBI scenario we assume that all furloughed workers would have lost their job, making most of them eligible to the unemployment benefit JSA or means-tested benefit UC.

Figure 6 shows the change in household net income by decile group of pre-crisis incomes for the following two scenarios: a) with a UBI, instead of the emergency measures (depicted by a hollow square); and b) with the emergency measures (a black circle, identical to the results in Fig. 1).

With both the UBI and the emergency measures the loss to net income would be close to 7% on average.[14] However, if we look along the distribution, we find that a UBI would have led to relatively large gains in net income at the bottom of the distribution: of 15.9% in decile 1, 5.5% in decile 2 and 1.8% in decile 3, compared to no change in deciles 1 and 2 and a small loss in decile 3 with emergency measures. However, these gains due to UBI at the bottom of the distribution would have been compensated by larger income reductions in deciles 6 to 10, compared to those with the emergency policies.

Among household types (Fig. B.8), the gainers from a UBI compared to the emergency measures would have been households with no earners, disabled individuals or elderly, those living in social housing or women in single person households. Households with elderly or disabled individuals would have in fact seen their incomes increasing with a UBI, while they saw their net income falling in the scenario with the emergency policies, on average. But the main losers from a UBI would have been households with two-and-more earners (located primarily in the upper half of the distribution) whose incomes would not have been as protected as with the emergency policies. Households with children and those renting privately would have also been worse-off, on average, with a UBI than with the emergency measures.

[13] In the UBI scenario, we also abolish the withdrawal of the existing Child Benefit (of £21.05 for the first child and £13.95 for any other child) for higher-income families to make the benefit universal but the income effects of this change are negligible.

[14] While on average the change in *non-equivalised* net income is the same in both scenarios, the change in *equivalised* net income differs slightly due to differences in the composition and size of families who receive UBI versus the emergency measures.

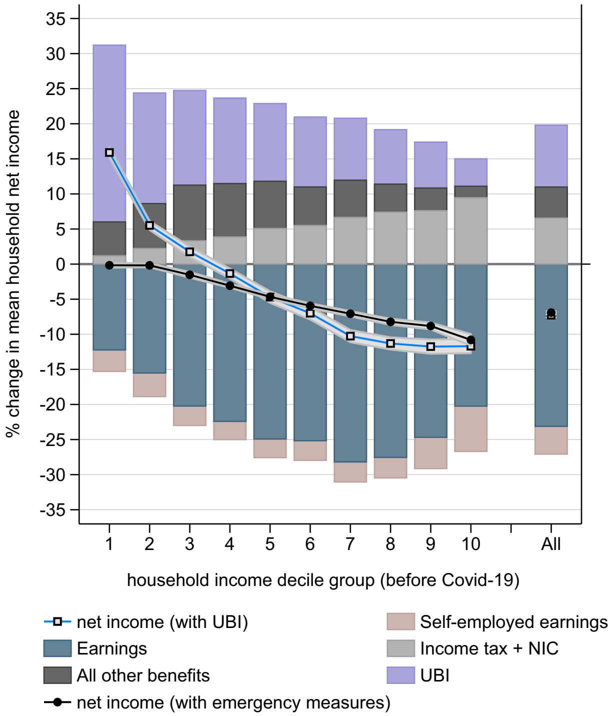

**Fig. 6** The impact of a Universal Basic Income (UBI) on household net incomes. *Notes:* The figure shows the potential distributional impact of a UBI. The results for the change in net income (with emergency measures) are the same as those shown in Fig. 1. Changes in income based on equivalised household net income. *Source:* Own calculations using UKMOD and FRS

In summary, compared to the emergency measures, there would have been different gainers and losers from the introduction of a UBI. A UBI would have benefited substantially also households not at all or less affected by the crisis, so overall it would have been less targeted at those hit hardest by the labour market shocks. On the other hand, different vulnerable groups would have benefited from a UBI.

Finally, our UBI simulation is based on what the government has been prepared to spend in emergency but this is far more than what it would spend in good times if a UBI is to be maintained in a post-pandemic world. In particular, the crisis is simulated to have worsened the government's fiscal position — revenues minus spending — by £16.3 billion per month in April and May 2020 (Table 4), where the emergency measures account for £12.2 billion per month. In other words, maintaining the UBI level we simulate after the pandemic while keeping the rest of the tax-benefit system intact will cost a lot. To make it affordable in the long-run, one would need to consider a different UBI which replaces at least partly existing benefits and is likely to need additional funding through tax rises. Designing a long-term UBI scheme would require re-thinking of the welfare state and making trade-offs between fiscal affordability, adequacy of a UBI to meet needs and securing the advantages of a UBI as a simplification of existing benefit programmes (Martinelli 2020).

## 5 Conclusions

Governments across the globe have taken drastic measures to address the economic costs of Covid-19. The UK government introduced income protection in the form of earnings subsidies for workers, and raised the level of means-tested state benefits. Families experiencing income losses due to Covid-19 can rely on state support through these measures as well as through the built-in automatic stabilisation response of the tax-benefit system.

Combining different sources of household micro-data and a tax-benefit model, we estimate the fiscal and distributional impact of Covid-19 during April and May 2020. We assess separately the impact of earnings losses, government's earnings subsidies (the CJRS and SEISS), state benefit increases and tax-benefit automatic stabilisers.

The Covid-19 pandemic caused a substantial shock to household incomes. We simulate that UK households, on average, sustained income losses in net income of 6.9%. Earned income fell by a substantial 12.6% of baseline net income. But policies protected household incomes to a substantial degree. Earnings subsidies for furloughed workers in particular played a major role in protecting household incomes, accounting for 13.2% of baseline net income. They supported households across the entire income distribution and provided the main insurance mechanism against the negative income shocks. The SEISS grant also provided small income gains of 1.3% on average.

Besides the earnings subsidies, tax-benefit policies provided needed income protection for families. At the bottom of the distribution, UC as a stabiliser helped families cope with the shocks. Covid-related increases to UC payments were especially targeted at the poorest decile, contributing to a net income gain of 2.8%. The unemployment benefit JSA also helped mitigate income losses, providing insurance against the shocks across the entire distribution. But the tax system itself also cushioned the shock, and automatic reductions in income tax and national insurance contributions were worth 4.1%, on average, of the pre-Covid baseline income, with the middle and top of the distribution benefiting mostly from income tax and NIC stabilisers due to the progressivity of the tax schedule and NIC.

We also look at the impact of a near-decade of austerity measures on the UK safety net. Compared to the 2011 system, we show that the 2020 *pre-Covid* tax-benefit system would have been less effective in providing insurance against the shocks. The extra Covid benefit spending strengthened the safety net, so the 2020 *post-Covid* system provides higher level of income protection at the bottom half of the distribution than the 2011 system. But these protections are temporary measures and do not undo the impact of austerity over the years.

Finally, we explore what would have been the impact on household incomes if a budget-neutral UBI was introduced in place of the Covid emergency measures. We simulate that, compared to the emergency measures, a UBI would have supported the incomes of different vulnerable groups but would have provided less protection to those hit hardest by the shocks.

**Supplementary Information** The online version contains supplementary material available at https://doi.org/10.1007/s10888-021-09491-w.

**Acknowledgements** We are grateful to Holly Sutherland, Tom Lee, the editor and anonymous reviewers for insightful comments and suggestions. The results presented here are based on UKMOD version A1.6. UKMOD is maintained, developed and managed by the Institute for Social and Economic Research (ISER) at the University of Essex and we are indebted to Holly Sutherland, Paola De Agostini and the many others who have contributed to its development. The process of extending and updating UKMOD is financially supported by the Nuffield Foundation, based on the annual update of the UK component of EUROMOD funded by the European Union Programme for Employment and Social Innovation "EaSI" (2014-2020). We make use of micro-data from the Family Resources Survey made available by the Department of Work and Pensions and Understanding Society COVID-19 Study by ISER, University of Essex via the UK Data Service. The results and their interpretation are our own responsibility.

**Open Access** This article is licensed under a Creative Commons Attribution 4.0 International License, which permits use, sharing, adaptation, distribution and reproduction in any medium or format, as long as you give appropriate credit to the original author(s) and the source, provide a link to the Creative Commons licence, and indicate if changes were made. The images or other third party material in this article are included in the article's Creative Commons licence, unless indicated otherwise in a credit line to the material. If material is not included in the article's Creative Commons licence and your intended use is not permitted by statutory regulation or exceeds the permitted use, you will need to obtain permission directly from the copyright holder. To view a copy of this licence, visit http://creativecommons.org/licenses/by/4.0/.

## Supplementary Information

ESM 1 (DOCX 140 kb)
