# Supplementary material for: Did the UK policy response to Covid-19 protect household incomes?
Source: J Econ Inequal. 2021 Aug 6;19(3):433–58. doi: 10.1007/s10888-021-09491-w (PMC8345904; doi:10.1007/s10888-021-09491-w)
Supplement: Supplementary file 1 — (DOCX 140 kb) [file 10888_2021_9491_MOESM1_ESM.docx]

**Online Supplementary Materials**

# A Tables

## **Table A.1:** Multinomial logit of employment status in **April** 2020

|  | Reduced hours  & earnings | Furloughed | Out of work |
| --- | --- | --- | --- |
| Constant | -1.948 | -.120 | -6.422* |
|  | (2.722) | (.871) | (3.320) |
| Men | -.422 | -.633 | 9.506** |
|  | (2.895) | (1.360) | (4.312) |
| Age | .005 | .014 | .089 |
|  | (.059) | (.018) | (.058) |
| Men x Age | .016 | -.007 | -.175** |
| ***Household type*:** | (.061) | (.025) | (.081) |
| Single adult, no children | .368 | -.092 | .029 |
|  | (.376) | (.187) | (.554) |
| Single adult living with children | .590 | -.193 | -.204 |
|  | (.448) | (.190) | (.640) |
| Multiple adults, no children | .340 | -.002 | -.070 |
|  | (.398) | (.192) | (.546) |
| Multiple adults living with children | .071 | .149 | .124 |
| ***Earnings quintile*:** | (.422) | (.196) | (.658) |
| 1 | ref | ref | ref |
| 2 | -.159 | -.313* | -2.136*** |
|  | (.248) | (.173) | (.613) |
| 3 | -.806** | -.716*** | -1.171* |
|  | (.338) | (.184) | (.621) |
| 4 | -.867** | -1.230*** | -.627 |
|  | (.377) | (.196) | (.595) |
| 5 | -.497 | -1.786*** | -1.788*** |
| ***Sex x Hours in work*:** | (.385) | (.219) | (.637) |
| Women x less than 20 | ref | ref | ref |
| Women x 20-34 | -.692*** | -.187 | -.747* |
|  | (.264) | (.203) | (.447) |
| Men x 20-34 | -.369 | 1.398** | -.160 |
|  | (.828) | (.609) | (.840) |
| Women x 35+ | -.934*** | -.349 | -.904 |
|  | (.343) | (.234) | (.632) |
| Men x 35+ | -.754 | 1.264** | -.510 |
| ***Sector*:** | (.616) | (.567) | (1.028) |
| Agriculture, Mining, Manufacture, Utilities | ref | ref | ref |
| Construction and Real Estate | -8.161** | -.421 | 2.081 |
|  | (4.098) | (1.449) | (3.484) |
| Wholesale and Retail Trade | -2.520 | -.838 | 7.298** |
|  | (2.892) | (1.000) | (3.470) |
| Transportation and Storage | 1.923 | 1.647 | -.453 |
|  | (5.230) | (1.853) | (3.972) |
| Accommodation and Food | -.163 | .473 | 6.314* |
|  | (2.919) | (1.122) | (3.584) |
| Information and Communication | 4.024 | -.950 | 5.539 |
|  | (3.333) | (2.355) | (3.895) |
| Financial and Insurance Activities | -.800 | -.148 | 15.868*** |
|  | (3.037) | (1.934) | (4.756) |
| Professional, Scientific and Technical Activities | .894 | .332 | 8.801** |
|  | (3.200) | (1.203) | (4.104) |
| Administrative and Support Service | -4.514 | .142 | -10.077*** |
|  | (3.311) | (1.488) | (3.376) |
| Public Administration and Defence | -1.383 | -.922 | 1.699 |
|  | (4.043) | (1.646) | (6.075) |
| Education | 1.012 | -.800 | 4.981 |

(base outcome = no drop in earnings)

|  | (2.703) | | (1.002) | | (3.697) | |
| --- | --- | --- | --- | --- | --- | --- |
| Human Health and Social Work | -.126 | | .045 | | 1.046 | |
|  | (2.817) | | (1.014) | | (3.534) | |
| Arts, Entertainment, Recreation and Other | 3.427 | | 3.496*** | | 7.721** | |
| ***Sex x Sector*:** | (3.323) | | (1.232) | | (3.713) | |
| Women x Agriculture, Mining, Manufacture, Utilities | ref | | ref | | ref | |
| Men x Construction and Real Estate | 12.097*** | | 3.530 | | -8.100* | |
|  | (4.455) | | (2.176) | | (4.911) | |
| Men x Wholesale and Retail Trade | 2.367 | | 1.445 | | -9.201* | |
|  | (3.232) | | (1.512) | | (4.759) | |
| Men x Transportation and Storage | -.141 | | 1.120 | | -4.528 | |
|  | (5.613) | | (2.257) | | (5.416) | |
| Men x Accommodation and Food | 2.932 | | 3.810** | | -8.809* | |
|  | (3.456) | | (1.821) | | (5.315) | |
| Men x Information and Communication | -5.921 | | -1.330 | | -13.164*** | |
|  | (4.304) | | (3.211) | | (4.659) | |
| Men x Financial and Insurance Activities | .145 | | -.097 | | -32.385*** | |
|  | (4.518) | | (2.809) | | (11.899) | |
| Men x Professional, Scientific and Technical Activities | 1.898 | | -1.881 | | -22.689*** | |
|  | (3.959) | | (1.912) | | (5.302) | |
| Men x Administrative and Support Service | 7.266** | | 1.641 | | 3.286 | |
|  | (3.676) | | (2.264) | | (4.447) | |
| Men x Public Administration and Defence | 3.099 | | .603 | | -9.862 | |
|  | (5.021) | | (2.658) | | (7.007) | |
| Men x Education | 1.046 | | -1.151 | | -9.151* | |
|  | (3.104) | | (1.972) | | (4.738) | |
| Men x Human Health and Social Work | -.691 | | .073 | | 19.547 | |
|  | (3.337) | | (1.913) | | (15.028) | |
| Men x Arts, Entertainment, Recreation and Other | -1.776 | | -2.179 | | -26.624*** | |
| ***Age x Sector*:** | (3.707) | | (1.862) | | (4.592) | |
| Age x Agriculture, Mining, Manufacture, Utilities | ref | | ref | | ref | |
| Age x Construction and Real Estate | .143* | | .002 | | -.046 | |
|  | (.080) | | (.032) | | (.061) | |
| Age x Wholesale and Retail Trade | .043 | | .011 | | -.139** | |
|  | (.063) | | (.023) | | (.066) | |
| Age x Transportation and Storage | -.033 | | -.047 | | .001 | |
|  | (.111) | | (.041) | | (.073) | |
| Age x Accommodation and Food | .023 | | .010 | | -.098 | |
|  | (.064) | | (.026) | | (.068) | |
| Age x Information and Communication | -.072 | | .014 | | -.072 | |
|  | (.077) | | (.051) | | (.072) | |
| Age x Financial and Insurance Activities | .007 | | -.024 | | -.494*** | |
|  | (.068) | | (.042) | | (.134) | |
| Age x Professional, Scientific and Technical Activities | -.030 | | -.013 | | -.207** | |
|  | (.070) | | (.026) | | (.091) | |
| Age x Administrative and Support Service | .083 | | -.005 | | -.080 | |
|  | (.070) | | (.032) | | (.062) | |
| Age x Public Administration and Defence | .018 | | -.029 | | -.039 | |
|  | (.084) | | (.035) | | (.115) | |
| Age x Education | -.030 | | -.005 | | -.091 | |
|  | (.059) | | (.021) | | (.068) | |
| Age x Human Health and Social Work | .000 | | -.035 | | -.025 | |
|  | (.061) | | (.022) | | (.065) | |
| Age x Arts, Entertainment, Recreation and Other | -.062 | | -.063** | | -.140** | |
| ***Sex x Age x Sector*:** | (.073) | | (.026) | | (.068) | |
| Women x Age x Agriculture, Mining, Manufacture, U | ref | | ref | | ref | |
| Men x Age x Construction and Real Estate | -.222** | | -.054 | | .169* | |
|  | (.087) | | (.046) | | (.093) | |
| Men x Age x Wholesale and Retail Trade | -.058 | | -.030 | | .159* | |
|  | (.070) | | (.033) | | (.096) | |
| Men x Age x Transportation and Storage | -.004 | | -.016 | | .096 | |
|  | (.119) | | (.048) | | (.105) | |
| Men x Age x Accommodation and Food | -.040 | | -.075* | | .144 | |
|  | (.076) | | (.041) | | (.119) | |
| Men x Age x Information and Communication | .092 | | -.018 | | .118 | |
|  | (.096) | | (.068) | | (.090) | |
| Men x Age x Financial and Insurance Activities | -.006 | | -.024 | | .793*** | |
|  | (.099) | | (.061) | | (.239) | |
| Men x Age x Professional, Scientific and Technical Activities | -.037 | | .029 | | .443*** | |
|  | (.087) | | (.040) | | (.117) | |
| Men x Age x Administrative and Support Service | | -.117 | | -.063 | | .191** |
|  | | (.082) | | (.051) | | (.089) |
| Men x Age x Public Administration and Defence | | -.083 | | .004 | | .183 |
|  | | (.106) | | (.059) | | (.134) |
| Men x Age x Education | | -.036 | | .010 | | .151 |
|  | | (.067) | | (.039) | | (.093) |
| Men x Age x Human Health and Social Work | | -.017 | | -.024 | | -.767 |
|  | | (.071) | | (.042) | | (.545) |
| Men x Age x Arts, Entertainment, Recreation and Other | | .001 | | .019 | | .209** |
|  | | (.077) | | (.039) | | (.090) |
| N | | 6122 | |  | |  |

*(table continued)* Multinomial logit of employment status in **May** 2020

(base outcome = no drop in earnings)

|  | Reduced hours  & earnings | Furloughed | Out of work |
| --- | --- | --- | --- |
| Constant | -2.491 | -.181 | -13.032*** |
|  | (1.549) | (1.096) | (2.312) |
| Men | 1.023 | -1.415 | 12.855*** |
|  | (2.037) | (1.702) | (3.281) |
| Age | .008 | .014 | .197*** |
|  | (.033) | (.022) | (.040) |
| Men x Age | .011 | -.007 | -.236*** |
| ***Household type*:** | (.041) | (.030) | (.056) |
| Single adult, no children | -.091 | .521** | .598 |
|  | (.279) | (.210) | (.498) |
| Single adult living with children | .262 | .445** | -1.134* |
|  | (.248) | (.214) | (.617) |
| Multiple adults, no children | -.073 | .512** | .283 |
|  | (.297) | (.234) | (.521) |
| Multiple adults living with children | -.252 | .588** | 1.040 |
| ***Earnings quintile*:** | (.326) | (.246) | (.639) |
| 1 | ref | ref | ref |
| 2 | .335 | -.137 | -.542 |
|  | (.263) | (.232) | (.370) |
| 3 | -.079 | -.407 | -.496 |
|  | (.323) | (.253) | (.489) |
| 4 | -.507 | -.625** | -.503 |
|  | (.331) | (.257) | (.525) |
| 5 | .104 | -1.232*** | -.742 |
| ***Sex x Hours in work*:** | (.309) | (.294) | (.501) |
| Women x less than 20 | ref | ref | ref |
| Women x 20-34 | .241 | -.249 | -1.409*** |
|  | (.315) | (.246) | (.378) |
| Men x 20-34 | -1.650* | 1.566* | .723 |
|  | (.842) | (.847) | (1.286) |
| Women x 35+ | .192 | -.497* | -2.203*** |
|  | (.367) | (.295) | (.517) |
| Men x 35+ | -1.085* | 1.460* | -.553 |
| ***Sector*:** | (.615) | (.826) | (1.275) |
| Agriculture, Mining, Manufacture, Utilities | ref | ref | ref |
| Construction and Real Estate | -7.217** | -1.819 | 9.513*** |
|  | (3.239) | (1.721) | (2.610) |
| Wholesale and Retail Trade | -2.437 | -1.509 | 13.781*** |
|  | (1.962) | (1.225) | (2.402) |
| Transportation and Storage | -21.692*** | .448 | 8.226 |
|  | (6.438) | (1.973) | (8.321) |
| Accommodation and Food | -1.905 | .090 | 12.381*** |
|  | (1.923) | (1.390) | (2.722) |
| Information and Communication | -2.643 | -4.255** | 9.428** |
|  | (2.061) | (2.143) | (4.595) |
| Financial and Insurance Activities | -1.243 | -.835 | 12.646*** |
|  | (1.982) | (2.419) | (3.626) |

| Professional, Scientific and Technical Activities | 1.923 | | .608 | 13.689*** | |
| --- | --- | --- | --- | --- | --- |
|  | (2.033) | | (1.458) | (2.638) | |
| Administrative and Support Service | .934 | | .378 | -2.475 | |
|  | (2.477) | | (1.558) | (2.439) | |
| Public Administration and Defence | -.522 | | -3.334* | 9.327* | |
|  | (2.311) | | (1.756) | (5.122) | |
| Education | .346 | | -1.328 | 13.103*** | |
|  | (1.658) | | (1.301) | (2.873) | |
| Human Health and Social Work | -2.295 | | -.453 | 12.241*** | |
|  | (2.002) | | (1.291) | (2.257) | |
| Arts, Entertainment, Recreation and Other | -1.981 | | 2.666* | 15.538*** | |
| ***Sex x Sector*:** | (3.527) | | (1.517) | (2.873) | |
| Women x Agriculture, Mining, Manufacture, Utilities | ref | | ref | ref | |
| Men x Construction and Real Estate | 9.346** | | 3.622 | -16.567*** | |
|  | (3.646) | | (2.312) | (3.628) | |
| Men x Wholesale and Retail Trade | .791 | | 1.342 | -16.268*** | |
|  | (2.699) | | (1.857) | (3.569) | |
| Men x Transportation and Storage | 21.571*** | | .722 | -17.409* | |
|  | (7.027) | | (2.470) | (9.926) | |
| Men x Accommodation and Food | -16.206*** | | 2.187 | -10.007** | |
|  | (2.528) | | (2.136) | (4.238) | |
| Men x Information and Communication | 1.709 | | 2.583 | -6.475 | |
|  | (2.846) | | (3.281) | (6.260) | |
| Men x Financial and Insurance Activities | -7.973*** | | -1.973 | -18.664*** | |
|  | (2.803) | | (3.004) | (6.967) | |
| Men x Professional, Scientific and Technical Activities | -1.767 | | -3.172 | -24.255*** | |
|  | (2.674) | | (2.124) | (3.634) | |
| Men x Administrative and Support Service | 2.976 | | 4.560* | 1.044 | |
|  | (3.254) | | (2.496) | (3.729) | |
| Men x Public Administration and Defence | 2.249 | | 1.392 | -13.955** | |
|  | (2.986) | | (3.527) | (5.902) | |
| Men x Education | .439 | | -.161 | -21.322*** | |
|  | (2.358) | | (2.318) | (6.169) | |
| Men x Human Health and Social Work | .056 | | -2.992 | -6.418 | |
|  | (4.075) | | (2.476) | (5.239) | |
| Men x Arts, Entertainment, Recreation and Other | -16.722 | | -3.161 | -33.847*** | |
| ***Age x Sector*:** | (13.953) | | (2.393) | (3.692) | |
| Age x Agriculture, Mining, Manufacture, Utilities | ref | | ref | ref | |
| Age x Construction and Real Estate | .135** | | .024 | -.165*** | |
|  | (.062) | | (.036) | (.046) | |
| Age x Wholesale and Retail Trade | .035 | | .019 | -.235*** | |
|  | (.042) | | (.027) | (.047) | |
| Age x Transportation and Storage | .393*** | | -.026 | -.119 | |
|  | (.118) | | (.043) | (.154) | |
| Age x Accommodation and Food | .058 | | .006 | -.177*** | |
|  | (.043) | | (.032) | (.052) | |
| Age x Information and Communication | .028 | | .070 | -.121 | |
|  | (.042) | | (.047) | (.091) | |
| Age x Financial and Insurance Activities | .018 | | -.047 | -.240*** | |
|  | (.043) | | (.059) | (.084) | |
| Age x Professional, Scientific and Technical Activities | -.046 | | -.028 | -.250*** | |
|  | (.044) | | (.031) | (.050) | |
| Age x Administrative and Support Service | -.002 | | -.016 | -.205*** | |
|  | (.052) | | (.035) | (.048) | |
| Age x Public Administration and Defence | -.012 | | .009 | -.164* | |
|  | (.050) | | (.036) | (.097) | |
| Age x Education | -.004 | | -.011 | -.225*** | |
|  | (.036) | | (.027) | (.053) | |
| Age x Human Health and Social Work | .045 | | -.032 | -.210*** | |
|  | (.044) | | (.027) | (.042) | |
| Age x Arts, Entertainment, Recreation and Other | .021 | | -.056* | -.294*** | |
| ***Sex x Age x Sector*:** | (.069) | | (.032) | (.064) | |
| Women x Age x Agriculture, Mining, Manufacture, U | ref | | ref | ref | |
| Men x Age x Construction and Real Estate | -.185*** | | -.056 | .276*** | |
|  | (.070) | | (.048) | (.062) | |
| Men x Age x Wholesale and Retail Trade | -.017 | | -.024 | .271*** | |
|  | (.059) | | (.040) | (.076) | |
| Men x Age x Transportation and Storage | -.390*** | | -.008 | .280 | |
|  | (.131) | | (.051) | (.184) | |
| Men x Age x Accommodation and Food | -.025 | | -.021 | .191** | |
|  | | (.055) | (.046) | | (.083) |
| Men x Age x Information and Communication | | -.031 | -.083 | | -.000 |
|  | | (.059) | (.066) | | (.149) |
| Men x Age x Financial and Insurance Activities | | .117* | .046 | | .346** |
|  | | (.061) | (.067) | | (.144) |
| Men x Age x Professional, Scientific and Technical Activities | | .048 | .067 | | .429*** |
|  | | (.057) | (.044) | | (.072) |
| Men x Age x Administrative and Support Service | | -.056 | -.112** | | .206*** |
|  | | (.071) | (.054) | | (.076) |
| Men x Age x Public Administration and Defence | | -.039 | -.009 | | .243** |
|  | | (.063) | (.076) | | (.110) |
| Men x Age x Education | | -.014 | .008 | | .363*** |
|  | | (.050) | (.047) | | (.110) |
| Men x Age x Human Health and Social Work | | -.033 | .047 | | .003 |
|  | | (.087) | (.051) | | (.157) |
| Men x Age x Arts, Entertainment, Recreation and Other | | .299 | .050 | | .338*** |
|  | | (.254) | (.050) | | (.079) |
| N | | 4954 |  | |  |

*Notes:* The dependent variable is employment status in April/May 2020. The model is estimated on the sample of employees, aged 20-63, with positive earnings from employment only in February 2020. The base outcome is *no drop in earnings*, i.e. of no more than £5 per week. The outcome *reduced hours and earnings* implies a fall of more than £5 in earnings and 1 working hour per week. Standard errors at a confidence level of 95% are shown in parenthesis. Significance levels indicated as * *p <* 0*.*1, ** *p <* 0*.*05, *** *p <* 0*.*01.

*Source:* Own calculations with Understanding Society COVID-19 data.

## **Table A.2:** Multinomial logit of self-employment status in **April** 2020

(base outcome = no drop in earnings)

|  | Reduced hours  & earnings | | Out of work | |
| --- | --- | --- | --- | --- |
| Constant | -1.059 | | -3.782** | |
|  | (.830) | | (1.899) | |
| Men | -.280 | | -1.575 | |
|  | (.438) | | (1.059) | |
| Age | -.004 | | .006 | |
| ***Household type*:** | (.011) | | (.025) | |
| Single adult, no children | .233 | | -.655 | |
|  | (.331) | | (.758) | |
| Single adult living with children | .229 | | -.562 | |
|  | (.332) | | (.756) | |
| Multiple adults, no children | .448 | | -1.112 | |
|  | (.325) | | (.815) | |
| Multiple adults living with children | .332 | | .475 | |
| ***Earnings ventile*:** | (.384) | | (.797) | |
| 1 | ref | | ref | |
| 2 | -.481 | | .169 | |
|  | (.481) | | (1.052) | |
| 3 | 1.254** | | 1.516 | |
|  | (.519) | | (1.235) | |
| 4 | .416 | | -.241 | |
|  | (.521) | | (1.464) | |
| 5 | .647 | | 1.506 | |
|  | (.637) | | (1.618) | |
| 6 | -.057 | | -14.304*** | |
|  | (.668) | | (1.218) | |
| 7 | .528 | | 2.311* | |
|  | (.572) | | (1.358) | |
| 8 | 1.256 | | -13.852*** | |
|  | (1.157) | | (1.610) | |
| 9 | .762 | | -14.031*** | |
|  | (.790) | | (1.279) | |
| 10 | 1.326** | | .908 | |
|  | (.580) | | (1.654) | |
| 11 | 1.289* | | 1.948 | |
|  | (.682) | | (1.319) | |
| 12 | .287 | | -13.840*** | |
|  | (.636) | | (1.234) | |
| 13 | | .384 | | -14.126*** |
|  | | (.724) | | (1.435) |
| 14 | | .283 | | -13.641*** |
|  | | (.545) | | (1.229) |
| 15 | | .082 | | 1.434 |
|  | | (.579) | | (1.801) |
| 16 | | 1.159* | | 2.689* |
|  | | (.625) | | (1.541) |
| 17 | | .416 | | -13.382*** |
|  | | (.683) | | (1.424) |
| 18 | | .868 | | 3.211** |
|  | | (.656) | | (1.499) |
| 19 | | .418 | | 1.540 |
|  | | (.562) | | (1.687) |
| 20 | | -.335 | | 1.818 |
| ***Sex x Hours in work*:** | | (.524) | | (1.352) |
| Women x less than 20 | | ref | | ref |
| Men x less than 20 | | -.341 | | -.658 |
|  | | (.512) | | (1.279) |
| Women x 20-34 | | .233 | | -1.009 |
|  | | (.432) | | (.991) |
| Men x 20-34 | | .480 | | .922 |
|  | | (.377) | | (.950) |
| Women x 35+ | | .014 | | -.049 |
| ***Sector*:** | | (.448) | | (1.143) |
| Agriculture, Mining, Manufacture, Utilities | | ref | | ref |
| Construction and Real Estate | | 1.933*** | | 2.531** |
|  | | (.478) | | (1.033) |
| Wholesale and Retail Trade | | 1.291*** | | .611 |
|  | | (.472) | | (1.475) |
| Transportation and Storage | | 1.347** | | -2.821** |
|  | | (.537) | | (1.363) |
| Accommodation and Food | | 3.787*** | | 4.080** |
|  | | (1.144) | | (1.644) |
| Information and Communication | | 1.205** | | 2.892*** |
|  | | (.577) | | (1.033) |
| Financial and Insurance Activities | | -.589 | | -15.483*** |
|  | | (.959) | | (1.138) |
| Professional, Scientific and Technical Activities | | .324 | | -.539 |
|  | | (.455) | | (1.392) |
| Administrative and Support Service | | 1.729*** | | 3.336*** |
|  | | (.537) | | (1.067) |
| Public Administration and Defence | | 1.126* | | -14.502*** |
|  | | (.670) | | (1.078) |
| Education | | .654 | | .548 |
|  | | (.452) | | (1.188) |
| Human Health and Social Work | | .830* | | -.857 |
|  | | (.445) | | (1.518) |
| Arts, Entertainment, Recreation and Other | | 1.676*** | | 1.166 |
|  | | (.472) | | (1.126) |
| N | | 1365 | |  |

*(table continued)* Multinomial logit of self-employment status in **May** 2020

(base outcome = no drop in earnings)

|  |  |  |
| --- | --- | --- |
|  |  |  |
|  |  |  |
|  |  |  |
|  |  |  |
|  |  |  |
|  |  |  |
|  |  |  |

|  | Reduced hours  & earnings | | Out of work | |
| --- | --- | --- | --- | --- |
| Constant | -.765 | | -17.133*** | |
|  | (1.028) | | (2.465) | |
| Men | -.625 | | -2.465* | |
|  | (.474) | | (1.353) | |
| Age | -.012 | | .017 | |
|  | (.012) | | (.037) | |
| ***Household type*:** |  | |  | |
| Single adult, no children | -.214 | | -.479 | |
|  | (.366) | | (.714) | |
| Single adult living with children | .167 | | -1.692 | |
|  | (.356) | | (1.325) | |
| Multiple adults, no children | -.192 | | -1.798* | |
|  | (.397) | | (1.012) | |
| Multiple adults living with children | -.681 | | -.656 | |
| ***Earnings ventile*:** | (.426) | | (.737) | |
| 1 | ref | | ref | |
| 2 | .009 | | -.180 | |
|  | (.505) | | (1.057) | |
| 3 | .463 | | -.525 | |
|  | (.541) | | (1.586) | |
| 4 | .681 | | 1.559 | |
|  | (.545) | | (1.326) | |
| 5 | .880 | | -13.742*** | |
|  | (.658) | | (1.390) | |
| 6 | .062 | | -15.467*** | |
|  | (.657) | | (1.453) | |
| 7 | .276 | | 3.012* | |
|  | (.904) | | (1.602) | |
| 8 | .804 | | 1.299 | |
|  | (.715) | | (1.629) | |
| 9 | .727 | | -14.601*** | |
|  | (.831) | | (1.086) | |
| 10 | 1.140** | | .340 | |
|  | (.577) | | (1.517) | |
| 11 | 1.227 | | 1.477 | |
|  | (.800) | | (1.962) | |
| 12 | 1.337* | | -13.446*** | |
|  | (.706) | | (1.666) | |
| 13 | 1.573* | | -11.738*** | |
|  | (.870) | | (1.653) | |
| 14 | .807 | | -13.703*** | |
|  | (.584) | | (1.464) | |
| 15 | 1.539** | | 1.722 | |
|  | (.732) | | (1.903) | |
| 16 | 1.791* | | -13.965*** | |
|  | (1.048) | | (1.972) | |
| 17 | 2.254*** | | 1.378 | |
|  | (.676) | | (2.086) | |
| 18 | .402 | | -15.490*** | |
|  | (.724) | | (1.292) | |
| 19 | 2.620*** | | 3.414* | |
|  | (.688) | | (2.024) | |
| 20 | 1.226* | | 3.231* | |
| ***Sex x Hours in work*:** | (.633) | | (1.716) | |
| Women x less than 20 | ref | | ref | |
| Men x less than 20 | .039 | | 3.100** | |
|  | (.584) | | (1.213) | |
| Women x 20-34 | .318 | | -1.177 | |
|  | (.471) | | (1.023) | |
| Men x 20-34 | 1.123*** | | 1.343 | |
|  | (.404) | | (.836) | |
| Women x 35+ | .142 | | -2.240* | |
| ***Sector*:** | (.456) | | (1.237) | |
| Agriculture, Mining, Manufacture, Utilities | ref | | ref | |
| Construction and Real Estate | .598 | | 16.229*** | |
|  | (.636) | | (1.017) | |
| Wholesale and Retail Trade | .827 | | 15.412*** | |
|  | (.653) | | (1.191) | |
| Transportation and Storage | .577 | | 15.618*** | |
|  | (.718) | | (1.104) | |
| Accommodation and Food | 2.081** | | 16.001*** | |
|  | (.811) | | (1.434) | |
| Information and Communication | .657 | | 15.146*** | |
|  | (.733) | | (1.149) | |
| Financial and Insurance Activities | -1.661 | | -2.864** | |
|  | (1.117) | | (1.367) | |
| Professional, Scientific and Technical Activities | .460 | | 13.804*** | |
|  | (.647) | | (1.505) | |
| Administrative and Support Service | 1.088 | | 15.957*** | |
|  | | (.709) | | (.907) |
| Public Administration and Defence | | .165 | | -1.335 |
|  | | (.738) | | (1.066) |
| Education | | .495 | | -.667 |
|  | | (.638) | | (1.201) |
| Human Health and Social Work | | .676 | | 13.987*** |
|  | | (.633) | | (1.388) |
| Arts, Entertainment, Recreation and Other | | 1.307** | | 14.593*** |
|  | | (.650) | | (1.031) |
| N | | 1074 | |  |

*Notes:* The dependent variable is self-employment status in April/May 2020. The model is estimated on the sample of self-employed, aged 20-63, with positive earnings from self-employment in February 2020. The base outcome is *no drop in earnings*, i.e. of no more than £5 per week. The outcome *reduced hours and earnings* implies a fall of more than £5 in earnings and 1 working hour per week. Significance levels indicated as * *p <* 0*.*1, ** *p <* 0*.*05, *** *p <* 0*.*01.

*Source:* Own calculations with Understanding Society COVID-19 data.

## **Table A.3:** Logit for receiving the SEISS grant in **May** 2020

| Constant | -1.633  (1.031) |
| --- | --- |
| Men | 1.153** (.446) |
| Age | .005  (.012) |

***Self-employment status in May vs Feb*:**

| No change | | ref |
| --- | --- | --- |
| With reduced hours and earnings | | .420*  (.229) |
| Unemployed  ***Household type*:** | | -.279  (.516) |
| Single adult, no children | | .393  (.468) |
| Single adult living with children | | .309  (.464) |
| Multiple adults, no children | | -.262  (.512) |
| Multiple adults living with children  ***Earnings ventile*:** | | .360  (.523) |
| 1 | | ref |
| 2 | | .286  (.562) |
| 3 | | -.083  (.589) |
| 4 | | .051  (.567) |
| 5 | | .287  (.734) |
| 6 | | -.069  (.670) |
| 7 | | -.483  (.799) |
| 8 | | -.074  (.710) |
| 9 | | -1.192  (1.028) |
| 10 | | .239  (.608) |
| 11 | | -1.009 (.696) |
| 12 | | -1.122 (.768) |
| 13 | | -1.284  (1.073) |
| 14 | | -.297  (.608) |
| 15 | | -.781  (.681) |
| 16 | -1.226 (.960) | |
| 17 | -.959  (.696) | |
| 18 | -.687  (.771) | |
| 19 | -.398  (.600) | |
| 20  ***Sex x Hours in work*:** | -1.446** (.600) | |
| Women x less than 20 | ref | |
| Men x less than 20 | -2.613*** (.621) | |
| Women x 20-34 | .678  (.461) | |
| Men x 20-34 | -.476  (.385) | |
| Women x 35+  ***Sector*:** | .603  (.447) | |
| Agriculture, Mining, Manufacture, Utilities | ref | |
| Construction and Real Estate | .838  (.580) | |
| Wholesale and Retail Trade | .417  (.599) | |
| Transportation and Storage | 1.609** (.698) | |
| Accommodation and Food | 1.223  (.913) | |
| Information and Communication | .370  (.613) | |
| Financial and Insurance Activities | .008  (1.011) | |
| Professional, Scientific and Technical Activities | .475  (.555) | |
| Administrative and Support Service | 1.053* (.580) | |
| Public Administration and Defence | -.252  (.828) | |
| Education | .443  (.582) | |
| Human Health and Social Work | .422  (.550) | |
| Arts, Entertainment, Recreation and Other | 1.255** (.578) | |
| N | 1074 | |

*Notes:* The model is estimated on the sample of self-employed, aged 20-63, with positive earnings from self-employment in February 2020. Standard errors at a confidence level of 95% are shown in parenthesis. Significance levels indicated as * *p <* 0*.*1, ** *p <* 0*.*05, *** *p <* 0*.*01.

*Source:* Own calculations with Understanding Society COVID-19 data.

## **Table A.4:** Household composition in the income distribution

|  | no earner 1 earner 2+ earners | | | with with with | | | with | lone mother |
| --- | --- | --- | --- | --- | --- | --- | --- | --- |
|  |  |  |  | children 3+ children disability | | | elderly |  |
| decile  1 | 22.3 | 9.7 | 1.6 | 9.6 | 18.4 | 6.9 | 12.0 | 15.2 |
| 2 | 15.7 | 12.2 | 2.5 | 12.6 | 22.6 | 11.2 | 12.1 | 21.8 |
| 3 | 14.6 | 11.9 | 4.5 | 12.2 | 17.4 | 13.9 | 13.3 | 20.3 |
| 4 | 12.8 | 10.6 | 7.0 | 11.9 | 11.4 | 19.5 | 12.4 | 14.0 |
| 5 | 10.2 | 11.2 | 8.7 | 10.9 | 8.3 | 17.3 | 11.7 | 10.5 |
| 6 | 8.1 | 9.7 | 11.6 | 10.4 | 5.5 | 13.9 | 10.6 | 7.2 |
| 7 | 5.2 | 9.4 | 14.1 | 9.6 | 4.7 | 8.1 | 8.2 | 6.2 |
| 8 | 4.1 | 8.1 | 15.7 | 8.3 | 4.5 | 4.5 | 7.3 | 1.9 |
| 9 | 3.7 | 8.3 | 16.6 | 7.0 | 3.1 | 3.0 | 6.3 | 1.9 |
| 10 | 3.3 | 8.7 | 17.8 | 7.4 | 4.1 | 1.6 | 6.1 | .9 |
| n households 9,826,848 7,949,500 10,049,547 7,801,746 1,292,746 3,679,514 8,597,443 1,661,870 | | | | | | | | |

*(table continued)*

|  | single-person hh: single-person hh: | |  | accommodation | |
| --- | --- | --- | --- | --- | --- |
|  | women | men | own | private rent social rent/other | |
| decile  1 | 18.9 | 17.6 | 9.5 | 10.2 | 18.4 |
| 2 | 14.0 | 10.3 | 7.9 | 10.5 | 16.7 |
| 3 | 13.3 | 12.2 | 7.2 | 11.3 | 19.6 |
| 4 | 13.4 | 10.6 | 8.2 | 11.5 | 15.1 |
| 5 | 10.8 | 9.9 | 9.5 | 10.5 | 11.2 |
| 6 | 9.6 | 8.2 | 10.1 | 10.2 | 8.5 |
| 7 | 6.7 | 7.7 | 11.0 | 9.3 | 5.2 |
| 8 | 4.8 | 6.5 | 11.3 | 9.4 | 2.9 |
| 9 | 3.9 | 8.5 | 12.1 | 9.1 | 1.7 |
| 10 | 4.6 | 8.7 | 13.3 | 8.0 | .8 |
| n households 4,308,600 3,795,195 17,687,422 5,112,757 5,025,716 | | | | | |

*Notes:* The table shows, for a given household type, what proportion (in %) of households are situated in each decile group. Income decile groups based on household equivalised net income before Covid-19. Each column refers to a household with certain characteristics. Household types for presence of earners are based on employment status before Covid-19. Household types can be overlapping.

*Source:* Own calculations with UKMOD and FRS.

## **Table A.5:** Change in the poverty gap in % points

|  | **Baseline** | **Impact of crisis** | |
| --- | --- | --- | --- |
|  | (in %) | (change to baseline)  fixed floating | |
| All | 4.164*** | .383*** | -.452*** |
|  | (.116) | (.043) | (.062) |
| Women | 4.194*** | .392*** | -.479*** |
|  | (.120) | (.045) | (.065) |
| Men | 4.133*** | .373*** | -.425*** |
|  | (.137) | (.047) | (.065) |
| Children | 4.980*** | .646*** | -.434*** |
| (.244) (.091) (.113) | | | |

*Notes:* The table shows the impact on the poverty gap of the employment and earnings shocks and the UK policy response, i.e. the *baseline* versus scenario *D*. The poverty gap is the mean shortfall of the total population from the poverty line (counting the non-poor as having zero shortfall), expressed in % of the poverty line. The poverty line is 60% of the median household equivalised net income in the baseline (fixed) or of the respective scenario (floating). Standard errors at a confidence level of 95% are shown in parenthesis. Bootstrapped standard errors after 200 replications. Significance levels indicated as * *p <* 0*.*1, ** *p <* 0*.*05, *** *p <* 0*.*01.

*Source:* Own calculations with UKMOD and FRS.

## **Table A.6:** Decomposing changes in income inequality

|  | **Baseline** | **Impact of crisis** (change to baseline) | | | | |
| --- | --- | --- | --- | --- | --- | --- |
|  | (in levels) | *E* + *K* | $S^{t}$ | $S^{b}$ | *P* | ∆ |
| Gini | .310*** | .005*** | .003*** | -.013*** | -.005*** | -.010*** |
|  | (.003) | (.002) | (.000) | (.000) | (.000) | (.001) |
| CV | .685*** | .003 | .007 | -.027*** | -.009*** | -.025* |
|  | (.029) | (.027) | (.010) | (.002) | (.000) | (.015) |
| MLD | .165*** | .004 | .003 | -.011*** | -.005*** | -.010*** |
|  | (.004) | (.003) | (.002) | (.002) | (.000) | (.001) |
| TI | .169*** | .005 | .002 | -.012*** | -.005*** | -.010*** |
| (.006) (.005) (.002) (.001) (.000) (.003) | | | | | | |

*Notes:* Inequality estimates based on equivalised household income. The total change ∆ is decomposed into: the contribution of earnings changes (*E* + *K*), tax/NIC and benefit automatic stabilisers ($S^{t}$ and $S^{b}$), and Covid-related benefit increases (*P*). CV=coefficient of variation; MLD=mean log deviation; TI=Theil index. Standard errors at a confidence level of 95% are shown in parenthesis. Bootstrapped standard errors after 200 replications. Significance levels indicated as * *p <* 0*.*1, ** *p <* 0*.*05, *** *p <* 0*.*01.

*Source:* Own calculations with UKMOD and FRS.

# B Figures

## **Figure B.1:** Impact of the crisis and policy response to Covid-19 on mean net income by decile: **households with 1 earner**

*Notes:* The figure shows the distributional impact of the employment and earnings shocks and the UK policy response, i.e. the *baseline* versus scenario *D*. Changes in net income are broken down by income source. Additional results for the change in net income in the absence of the Covid emergency measures (i.e. CJRS, SEISS and increases to UC and other means-tested benefits) are also shown. Changes in income based on equivalised household net income. All-population deciles.

*Source:* Own calculations using UKMOD and FRS.

## **Figure B.2**: Impact of the crisis and policy response to Covid-19 on mean net income by decile: **households with 2+ earners**

*Notes:* The figure shows the distributional impact of the employment and earnings shocks and the UK policy response, i.e. the *baseline* versus scenario *D*. Changes in net income are broken down by income source. Additional results for the change in net income in the absence of the Covid emergency measures (i.e. CJRS, SEISS and increases to UC and other means-tested benefits) are also shown. Changes in income based on equivalised household net income. All-population deciles.

*Source:* Own calculations using UKMOD and FRS.

## **Figure B.3:** Impact of the crisis and policy response to Covid-19 on mean net income by industry

*Notes:* The figure shows the distributional impact of the employment and earnings shocks and the UK policy response, i.e. the *baseline* versus scenario *D*, for the sample of workers with positive earnings from (self-)employment in the baseline (before Covid-19). Changes in net income are broken down by income source. Additional results for the change in net income in the absence of the Covid emergency measures (i.e. CJRS, SEISS and increases to UC and other means-tested benefits) are also shown. Changes in income based on equivalised household net income. Thus, the effects by industry partly depend on household composition. For example, if the crisis led to income losses among individuals in “Arts, Entertainment, Recreation and Other”, we will observe income losses for this group and for the members of their households, who may be working in industries less affected by the crisis, because of the equal household income sharing rule we apply.

*Source:* Own calculations using UKMOD and FRS.

## **Figure B.4:** Impact of the crisis and policy response to Covid-19 on mean net income by decile and tax-benefit policy: **households with 1 earner**

*Notes:* Left plot shows impact of automatic stabilisers, i.e. the *baseline* versus scenario *C*. Right plot shows impact of Covid-related benefit increases, i.e. scenario *C* versus *D*. Changes in total net income and the contribution of earnings changes, CJRS subsidies and SEISS grant are omitted. Changes in income based on equivalised household net income. Other benefits include the Council Tax Reduction, Child Tax Credit, Income support, income-related Employment and Support Allowance, income-based JSA, Pension Credit, Scottish benefits (Sure Start Maternity Grant and Best Start Grant). No simulations to Statutory Sickness Pay.

*Source:* Own calculations using UKMOD and FRS.

## **Figure B.5:** Impact of the crisis and policy response to Covid-19 on mean net income by decile and tax-benefit policy: **households with 2+ earners**

*Notes:* Left plot shows impact of automatic stabilisers, i.e. the *baseline* versus scenario *C*. Right plot shows impact of Covid-related benefit increases, i.e. scenario *C* versus *D*. Changes in total net income and the contribution of earnings changes, CJRS subsidies and SEISS grant are omitted. Changes in income based on equivalised household net income. Other benefits include the Council Tax Reduction, Child Tax Credit, Income support, income-related Employment and Support Allowance, income-based JSA, Pension Credit, Scottish benefits (Sure Start Maternity Grant and Best Start Grant). No simulations to Statutory Sickness Pay.

*Source:* Own calculations using UKMOD and FRS.

## **Figure B.6:** Impact of the crisis and policy response to Covid-19 on mean net income by household type

*Notes:* The figure shows the distributional impact of the employment and earnings shocks and the UK policy response, i.e. the *baseline* versus scenario *D*. Changes in net income are broken down by income source. Additional results for the change in net income in the absence of the Covid emergency measures (i.e. CJRS, SEISS and increases to UC and other means-tested benefits) are also shown. Changes in income based on equivalised household net income. Household types for presence of earners are based on employment status before Covid-19.

*Source:* Own calculations using UKMOD and FRS.

## **Figure B.7:** Impact of the crisis and policy response to Covid-19 on mean net income by household type and tax-benefit policy

*Notes:* Left plot shows impact of automatic stabilisers, i.e. the *baseline* versus scenario *C*. Right plot shows impact of Covid-related benefit increases, i.e. scenario *C* versus *D*. Changes in total net income and the contribution of earnings changes, CJRS subsidies and SEISS grant are omitted. Changes in income based on equivalised household net income. Household types for presence of earners are based on employment status before Covid-19. Other benefits include the Council Tax Reduction, Child Tax Credit, Income support, income-related Employment and Support Allowance, income-based JSA, Pension Credit, Scottish benefits (Sure Start Maternity Grant and Best Start Grant). No simulations to Statutory Sickness Pay.

*Source:* Own calculations using UKMOD and FRS.

## **Figure B.8:** Impact of a Universal Basic Income (UBI) on mean net income by household type

*Notes:* The figure shows the potential distributional impact of a UBI. The results for the change in net income (with emergency measures) are the same as those shown in Figure B.6. Changes in income based on equivalised household net income. Household types for presence of earners are based on employment status before Covid-19.

*Source:* Own calculations using UKMOD and FRS.

## **Figure B.9:** Impact of the crisis and policy response to Covid-19 on mean net income by age group

*Notes:* The figure shows the distributional impact of the employment and earnings shocks and the UK policy response, i.e. the *baseline* versus scenario *D*. Changes in income based on equivalised household net income.

*Source:* Own calculations using UKMOD and FRS.

# C Simulation of the CJRS subsidy and SEISS grant

Employees who are predicted to be furloughed receive the CJRS subsidy in April and May. To calculate how much workers are entitled to under the scheme, we use information on (baseline) total gross earnings from employment (FRS variable *inearns*). Self-employed predicted to be entitled to the SEISS grant receive the subsidy in May only (when it was first paid out). We estimate the size of the grant as if it was made on a monthly basis (rather than the actual size worth three months of profits) and base our calculations on

(baseline) total gross self-employed earnings (FRS variable *seincam2*).^^[[1]](#footnote-1)^^

# D Benefit take-up

The calculations of means-tested benefits and tax credits in UKMOD account for non-take-up. Take-up rates are based on the 2017 mid-point estimates on a caseload basis by the Department for Work and Pensions and HM Revenue and Customs.^^[[2]](#footnote-2)^^ Households from the FRS sample are randomly selected to take-up their simulated entitlements so that the number of takers is in line with the official take-up rates. For UC, there is no evidence yet for the extent of non-take-up. However, due to the means-tested nature of the benefit, it is plausible that, like the benefits UC is replacing, UC does not reach all entitled families. A take-up rate of 87% is assumed for UC (as the take-up rate estimated for the Income Support benefit for families without children). For more information on the take-up assumptions in UKMOD, see Reis & Tasseva (2020).

In scenarios *C* and *D*, we assume that families affected by the shocks do not take up UC if they were entitled to and did not take up UC prior to the shock. That is their take-up behaviour remains the same even after the change in their circumstances. In the case of affected families who become newly entitled to UC after the shock, the majority takes up UC but a small proportion of eligible families (as with Income Support) do not receive UC (e.g. due to errors in assessing their eligibility).

# References

Reis, S., & Tasseva, I.: UKMOD/EUROMOD Country Report: United Kingdom (UK) 2018–2024, CeMPA Working Paper 7/20, University of Essex, Colchester (2020)

1. In reality, the grant is calculated based on the average of last three-years profits and provided only to those with profits up to £50,000 per year. As we do not observe this information in the FRS and UKHLS data, we do not account for it in the simulations. [↑](#footnote-ref-1)
2. DWP 2017/18 take-up estimates for income-related benefits available here:

   https://www.gov.uk/government/statistics/income-related-benefits-estimates-of-take-up-financial-year-2017-to-2018. HMRC 2017/18 take-up estimates for tax credits available here: https://www.gov.uk/government/statistics/child-benefit-child-tax-credit-ctc-and-working-tax-credit-wtc-take-up-rates-2017-to-2018 [↑](#footnote-ref-2)
